# Dynamics and landscape of academic discourse on environmental attitudes and behaviors since the 1970s

**DOI:** 10.3389/fsoc.2023.1136972

**Published:** 2023-07-14

**Authors:** Audrone Telesiene, Markus Hadler

**Affiliations:** ^1^Civil Society and Sustainability Research Group, Kaunas University of Technology, Kaunas, Lithuania; ^2^Department of Sociology, University of Graz, Graz, Austria; ^3^Department of Sociology, Macquarie University, Sydney, NSW, Australia

**Keywords:** environmental attitudes, environmental behavior, systematic literature review, quantitative content analysis, academic discourse

## Abstract

**Introduction:**

This study addresses the lack of systematic review and analysis of the academic discourse on environmental attitudes and behaviors. Despite the wealth of knowledge published in academic journals, there is a need to understand the order and content of this discourse, including the employed theoretical approaches and empirical evidence.

**Methods:**

A combination of systematic literature review and quantitative content analysis methods was employed. Articles for analysis were identified through Web of Science and SCOPUS, followed by a detailed analysis of 200 papers from the journal Environment and Behavior. The study aimed to explore the historical stages, theoretical diversity, and the empirical evidence brought forward in the academic discourse on environmental attitudes and behaviors.

**Results:**

The findings reveal distinct historical stages within the academic field of environmental attitudes and behaviors. There is a notable growth in theoretical diversity and intensity of the discourse, particularly after 2000. The dominance of socio-psychological explanatory models is evident. Furthermore, the empirical evidence base is geographically limited, mostly coming from the US.

**Discussion:**

The study discusses the limitations of the academic discourse on environmental attitudes and behaviors and provides guidelines for future research. It emphasizes the need to address the identified shortcomings, such as expanding theoretical perspectives and increasing the geographical diversity of empirical evidence. The study's findings contribute to understanding the development and characteristics of the academic field, while also identifying avenues for further exploration.

## 1. Introduction

The scientific field of environmental sociology is counting its sixth decade already (Dunlap, 2017). Research on environmental attitudes and behaviors has proved itself as a fertile research tradition within the field (Milfont et al., 2019). The research tradition has produced some of the most influential papers that coined the New Ecological Paradigm (Catton and Dunlap, 1978; Dunlap et al., 2000), has served as a testbed for the development of post-materialism (Inglehart, 2015), Value-Belief-Norm (Stern et al., 1999), Planned Behavior (Ajzen, 1991) and other theories.

The number of different theories, assumptions, concepts brought forward, and empirical studies published since the 1970s is striking (Lachmann, 2016). Yet, we assume that these developments have inherent trends and patterns that resemble trajectories discussed concerning the general scientific progress (Kuhn, 2012) and the attention cycle of scientific issues (Downs, 1972). Using these two approaches as a backdrop, our endeavor aims to reflect critically upon the development of the academic discourse on environmental attitudes and behaviors across the globe and to explore and systemize the theoretical and methodological grounds that constitute this research tradition.

In contrast to review-articles that summarize and evaluate existing research (Dietz et al., 2005; Zhu et al., 2021), articles that combine different approaches (Stern, 2000), and meta-studies that aim to combine theories and results from different sources (Klöckner, 2013), we contribute to existing research by combining a systematic literature review and a quantitative content analysis of published papers to depict the frequency of different theories, their trajectory over time, their interconnectedness, and the methods and empirical evidence used in testing these theories.

As for the scope of our analysis, we started with the consideration of all texts on environmental attitudes and behaviors, recorded in Web of Science and Scopus. We then decided to focus on the journal *Environment and Behavior* (EB) as it is among the most frequent outlets in both scientific databases, deals specifically with the interaction between behaviors and the environment, has been published since 1969, and covers the field of environmental sciences basically from the beginning. Hence, our detailed analyses of all relevant papers published in EB can be considered an analysis of a typical case—a journal that has shaped research in this area for decades (Milfont et al., 2019; Zhu et al., 2021).

Our detailed analyses trace the occurrence and co-occurrence of theories focusing on environmental behaviors and attitudes, and the empirical data presented supporting the reported theories. We thus go beyond Milfont et al. (2019) bibliometric analysis of EB, which identified a distinct cluster of articles on environmental concern, sustainability, and pro-environmental behaviors, but did not have the room to analyze the dominant theories and their development. Yet, we are also aware of the limitations of our analyses, as we are only able to discuss the trajectory of these theories in very general terms, and cannot offer a detailed history of each theory within this paper.

In line with these considerations, our paper is organized as follows. The subsequent section situates our research in the literature on the development of scientific progress and presents our research questions in more detail. This part is followed by an overview of our sampling techniques and analytical approach. The results section starts with an enumeration of the mentioned theories, shows their development over time, their interconnectedness, and the empirical evidence used in the articles. The conclusions discuss the development of theories and used empirical evidence concerning Kuhn's views on scientific progress and Downs' attention cycle. They highlight a post-2000 shift toward a more frequent use of socio-psychological variables (as compared to socio-structural, situational and macro level variables), limitations in the geographical distribution of empirical evidence, and offer suggestions for further research.

## 2. Background and research questions

Kuhn (2012) described scientific progress as a step-wise process, where new paradigms become dominant when theories become less accurate. The shift to new paradigms is characterized by a phase of competing views, where some scholars defend old views, while the new paradigm gains ground in the scientific community, becomes dominant, and eventually turns into a coherent theory. Once a new paradigm is established, science enters a period of normal science, where research applies the new paradigm and gains additional insights. Eventually, however, the now dominant paradigm might get replaced by another paradigm.

We use a few well-known developments and publications in environmental social science to highlight the applicability of Kuhn's views. Environmental sciences in general gained momentum in the 1960s when the effects of modern societies on the environment as well as the negative counter-effects of the environment on societies became salient and the mutual dependency of humans and the environment was recognized. Catton and Dunlap (1978), for example, proposed the New Ecological Paradigm (NEP) emphasizing the mutual dependency of humans and the environment, which ought to replace the Human Exemptionalism Paradigm (HEP) and its assumption that human actions are exempt from environmental forces and purely cultural. Concurrently, theories such as ecological modernization, the theory of cultural value orientations, and other theories appeared (Lachmann, 2016; Harper and Snowden, 2017).

This period can be seen as the initial appearance of a new paradigm that is moving into various disciplines. Following Kuhn's logic, we would expect a period of competing views and discussions of the different effects until science reaches a new consensus. With a special focus on environmental behavior, we see such unifying efforts, for example, by Stern (2000), who attempted to develop a coherent theory of environmentally significant behavior and, more recently, by Klöckner (2013) on a comprehensive model of the psychology of environmental behavior. In Kuhn's terms, the period of normal science has not been reached yet, as we can still identify the rising of distinct lines of theoretical views.

Besides these developments, academic discourse (the same as media discourse) is also subject to issue attention cycles (Downs, 1972). It can be described through developmental dynamics, with attention cycles usually fluctuating and the attention span describing the distance between the peaks of the cycles. The issue attention cycle includes stages of latency, breakthrough, boom, and fatigue (Moher et al., 2015). Critical productivity of the discourse is usually reached in the boom stage. Following these ideas, we can also expect a dynamic development of theories related to environmental attitudes and behaviors.

The mentioning of NEP and HEP as well as of the other theories in the above paragraphs does not imply that our paper is limited to these specific views. We used them only as illustrative examples, whereas our analyses consider all articles that study environmental behaviors or attitudes, mention specifically a theory, and use empirical data.

As for the exploration of theoretical backgrounds of the articles of the academic discourse, we ask:

RQ1. What are the dynamics and the intensity of the academic discourse on environmental attitudes and behaviors;
RQ2. What chronological stages can be identified in the development of this academic discourse;
RQ3. Which theoretical approaches are mentioned within the theoretical frameworks of the reported empirical studies;
RQ4. How does the prevalence of different theories shift over time;
RQ5. How often do these theoretical assumptions co-occur in academic texts;
RQ6. Which theoretical assumptions co-occur most often.

Alongside the theoretical background, we also look into the character of the empirical support, which is reported in these academic texts. Here, we are specifically aware of the tension between the claim of universal applicability and the availability of only local or group-specific samples. As for the empirical background used in the analyzed texts, we ask:

RQ7. What is the geographical coverage of the reported empirical studies;
RQ8. What is the socio-structural coverage of the reported empirical studies (does it apply to societies in their entirety or only to some social groups);
RQ9. What is the chronology and recentness of the empirical studies;
RQ10. Which explanatory variables are used in the empirical studies?

## 3. Materials and methods

To answer the research questions, our study combined two methodological strategies: a systematic literature review and a quantitative content analysis enabled by MAXQDA Analytics Pro (2022).

### 3.1. Materials and sampling

Our analysis considers academic publications that report empirical research on environmental behaviors, attitudes, and related concerns. We followed the PRISMA-P 2015 statement (Preferred Reporting Items for Systematic Reviews and Meta-Analyses) for developing and reporting our sampling strategy (Moher et al., 2015). Hence, a systematic review protocol with a PRISMA Flow Diagram was drafted and is made available to ensure accountability, research integrity, and transparency of the completed review (see Appendix 1).

Texts eligible for inclusion were located in the Web of Science Core Collection and SCOPUS databases (Gusenbauer and Haddaway, 2020). Our sampling included several major steps. First, the total set of relevant academic texts was drawn through criteria sampling considering all texts up to the end of the year 2020.^1^ We limited the search results to articles, books, and book chapters from social sciences, environmental sciences, and multidisciplinary research. Our inclusion criteria are: the use of the keywords “environmental attitudes” and “environmental behavio(u)r” in titles, abstracts, or keywords; the type of document: article, book, book chapter; social sciences, environmental sciences, or multidisciplinary research indicated as the main subject area. Our exclusion criteria after reading the abstracts or texts are: if texts included theoretical discussion but did not report an empirical study; if texts were meta-analyses; if neither environmental attitudes nor individual environmental behavior was the dependent variables of the study. The second step employed typical case sampling because we aimed at studying typical trends found in the academic texts explaining individual environmental attitudes and behavior. To identify the most typical cases we analyzed the publication sources (journal and book titles). An analysis of main publication sources as returned by Web of Science Core Collection database showed that most of the texts were published in *Journal of Environmental Psychology (established in 1980), Environment and Behavior (est. in 1969), Journal of Cleaner Production (est. in 1993)*, and *Sustainability (est. in 2009)*. An analysis of the main publication sources returned by SCOPUS showed that most of the texts were published in *Journal of Environmental Management (est. 1973), Sustainability (est. in 2009), Environment and Behavior (est. in 1969), Journal of Environmental Management (est. in 1974)*, and *International Journal of Environmental Research and Public Health (est. in 2004)*. The results indicate that the journal *Environment and Behavior* (EB) is the most typical source, as it is the only source that appears among top sources in both databases, has a focus on the link between behaviors, attitudes, and the environment, and was established shortly after the inception of environmental sciences. Recent research (Zhu et al., 2021) further confirms that EB journal is the most popular journal in the field of environmental behavior studies. We thus can assume that work published in EB influences related research significantly across the globe and that an analysis of this journal is of interest to many scholars.

### 3.2. Coding procedures

The data for our in-depth analysis were derived by downloading all full texts from the EB journal that matched our initial inclusion criteria. Two hundred and twelve texts were considered initially; thereof 12 had to be excluded due to irrelevance or because they presented meta-analyses. Our final data thus consist of semi-automatically and manually coded excerpts from 200 full texts published in EB between 1971 and 2020, managed using MAXQDA Analytics Pro (2022). Coding and analysis approaches included: lexical search and automatic coding of text segments; refining automatic coding by manual coding of significant text passages; quantifying codes; sub-code statistics (frequency distribution of coded segments); use of complex coding queries; coding matrix analysis; code relations analysis (co-occurrence); and code mapping.

The texts were coded via a combination of inductive and deductive thinking. A deductive approach was used to develop the starting list of codes. Coding was refined and constantly adjusted according to the new information pieces discovered during a close reading of the articles (inductive thinking). An overview of our coding framework is presented in Tables 1, 2 (complete information on the coding system is available from the authors). The first version of the coding system was tested by coding 20 articles from the sample. The coding system was refined and further discussed several times to make sure that it results in reliable coding.^2^

**Table 1:** Coding scheme for the theoretical framework of the analyzed articles (total of 200 documents).

**Code family^a^**	**Codes**	**Occurrence (*n*)**
Theoretical approaches mentioned in the articles	Affluence and prosperity	10
	Attitude theory	5
	Campbell paradigm	4
	Cultural theory	5
	Dominant social paradigm	13
	Ecocentrism-technocentrism	7
	Environmentally significant behavior	6
	Gender Socialization theory	4
	Hofstede's theory of cultural dimensions	4
	Maslow's hierarchy of needs	4
	NIMBY	4
	New ecological paradigm (HEP-NEP)	65
	Norm activation theory	17
	Post-materialism	20
	Rational choice	5
	Risk society	3
	Self-perception theory	4
	Social dilemmas and game theory	6
	Social identity theory	6
	Social learning theory	8
	Theory of cultural value orientations (Schwartz)	15
	Theory of planned behavior (Ajzen)	31
	Theory of reasoned action	21
	Value orientations (Stern)	21
	Value-belief-norm (Stern)	18
	White's Judeo-Christian theory	4
	Other	125
Total (coded segments)		435

^a^Only a selection of codes is listed; full list available from authors.

**Table 2:** Coding scheme for the methodological characteristics of the analyzed articles (total of 200 documents).

**Code family^a^**	**Codes and sub-codes**	**Coded segments**	**Coded documents**
Methodological characteristics	Year of publication	200	200
	Year of empirical study	111	100
	Geographical coverage	471	191
	Target population (with sub-codes):	215	193
	Social group or organization	110	101
	Territory within country	68	63
	Country	27	25
	Multiple countries	10	10
	Representativeness (with sub-codes):	216	193
	Representative	76	69
	Non-representative	140	128
	Data source (with sub-codes):	214	193
	Original studies	180	163
	Regular national or regional studies	24	23
	International programs	10	10
	Main variables (with sub-codes):	1,695	185
	Dependent	397	177
	Explanatory	1,298	179
Total		3,122	200

^a^Only a selection of codes is listed; full list available from authors.

The first set of codes relates to RQ1-6 and codes the aspects of theoretical frameworks of the analyzed texts. We aimed to analyze which theoretical approaches were mentioned in the articles and how these were combined into a theoretical framework that precedes “analysis” or “results” chapters. Inspired by Lachmann (2016) and also leaning on the personal scholarly experience of the authors, a preliminary list of theoretical approaches including different theories of environmentalism such as rational choice, post-materialism, new ecological paradigm, ecological modernization, biographical availability theory, theory of planned behavior, and value-belief-norm theory. The list was open and refined inductively many times during the in-depth reading of the articles. Theoretical approaches were coded under different labels when a theory, or a thesis, or a paradigm was explicitly mentioned in a text, or when theory-specific keywords were used in an article (e.g., “biospheric” only pertains to the value orientations theory developed by Stern and colleagues). We thus did not pre-define what constitutes a theory but followed the subjective classifications of the authors of the analyzed papers.

Table 1 presents the final coding information for our theoretical framework. The most frequent code is the “New Ecological Paradigm (NEP),” which appeared in 65 documents out of a total of 200 documents. NEP has been historically conceptualized as a challenge to the Dominant Social Paradigm, but since Catton and Dunlap had two separate measures (multi-dimensional in case of Dominant Social Paradigm), they are often used separately. For a parallel analysis contrasting the NEP with the “Human Exemptionalism Paradigm” see Catton and Dunlap (1980).

The second most common code is the “Theory of planned behavior” which postulates that behavior is determined by intentions, which are influenced by an individual's attitudes, subjective norms, and perceived behavioral control. It is followed by “Postmaterialism,” a theory that postulates that people's values shift from materialistic concerns to postmaterialistic concerns that also entail pro-environmental views. The fourth most common label is the “Theory of Reasoned Action,” which emphasizes rational considerations and intentions, as well as attitudes toward the behavior and subjective norms. The fifth most common mentioning was “Value Orientations,” which focuses on individuals' underlying belief systems and values.

The second set of codes relates to RQ7-10 and refers to methodological characteristics of the empirical studies as reported in the analyzed texts. While trying to understand what kinds of empirical evidence are used to explain individual environmental attitudes and behaviors, we looked at the “methods” discussions in the articles. Our attention to the methodological aspects of the articles was spurred by Arbuckle et al. (2015, p. 210), who found a huge diversity of measures employed and stated that this makes a synthetic interpretation of results difficult.

Table 2 presents the coding scheme for the methodological characteristics of the analyzed articles.

The “geographical coverage sub-code” (see Table 2) shows where the empirical studies, as reported in the articles, were conducted. It is coded with labels of country names as used by the World Bank. “Year of the reported empirical study” indicates when the reported empirical studies were conducted. The socio-structural coverage of the empirical studies was coded as “Target population” and indicates whether the societies were researched in their entirety (sub-codes “country” or “multiple countries”) or if only some social groups, certain structures or territorial units within societies were covered (sub-codes of “social groups or organizations” or “territories within countries”). “Dependent variables” and the different mentioned “explanatory variables” as well as “data collection method” were openly coded. Texts indicating if the reported empirical research was representative were openly coded under “Representative=Yes/No” sub-codes. “Main focus” code was of instrumental value during conducting this analysis as it captures main questions discussed in an article in a single concise phrase. Sub-codes of the “Data source” parent code indicate if the data, as reported in the article, initially came from an original study conducted by the authors or rather came from national or international studies conducted by others.

## 4. Results

### 4.1. Development over time

**Figure 1:**
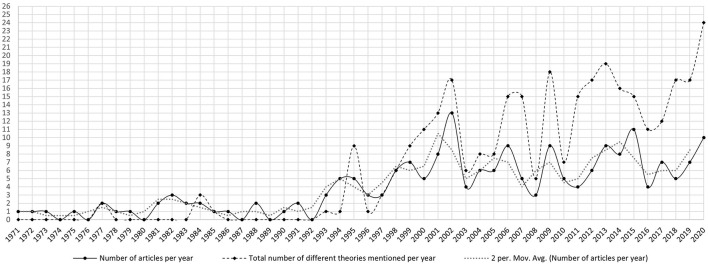
Yearly distribution of articles and different theories mentioned from 1971 to 2020.

Our first set of questions focuses on the development of the academic discourse over time. We are considering (RQ1) the dynamics and the intensity of the academic discourse on environmental attitudes and behaviors and (RQ2) if chronological stages can be identified in the development of this academic discourse. Figure 1 shows our results in this regard.

The yearly numbers of articles (continuous line in Figure 1) have been calculated to represent the dynamics and intensity of the academic discourse on environmental attitudes and behaviors. The mentioning of different theories within these articles (broken line in Figure 1) was summed up to represent the development of theoretical diversity within the field. The intensity and the content usually define the order of discourse (Jorgensen and Phillips, 2002). Fundamental changes in either or both of the criteria point to the changing discourse order and serves for the identification of major periods within a discourse. These changes in the reported numbers go beyond the increase of annual issues of EB over time—it started with two issues in 1969, then expanded to 3 in 1970, 4 in 1973, 6 in 1981, 8 in 2013, and 10 in 2015 (Milfont et al., 2019).

Our results point to several turning points. The first clear change in the discourse happened in 1993. This year is the cut-off point beyond which the number of articles on environmental attitudes and behaviors started to increase and did not drop below the pre-1993 level again. It should be noted that this increase was not fueled by a general increase in articles in the journal, as the general increase was noticed only a decade later (Milfont et al., 2019). In this instance, the increasing intensity of the discourse defined the change of discourse order.

The data in the graph also shows how often various theories have been mentioned in the analyzed articles. It does not show the birth of theories themselves, but rather, a theoretical fertilization of the academic discourse. The specific theories and how often they have been mentioned are addressed in Table 3.

**Table 3:** Theoretical approaches mentioned in the analyzed articles.

**Periods of publication:**	**Before 2000**		**2000 and later**		**Total**	
**Theoretical approaches:**	**% of all docs**	**Valid % of docs**	**% of all docs**	**Valid % of docs**	**% of all docs**	**Valid % of docs**
Attitude theory	1.8	5.9	3.0	3.9	2.6	4.2
Affluence and prosperity	1.8	5.9	6.0	7.8	4.7	7.6
Campbell paradigm	–	–	2.2	2.9	1.6	2.5
Cultural theory	1.8	5.9	2.2	2.9	2.1	3.4
Dominant social paradigm	1.8	5.9	9.0	11.8	6.8	10.9
Ecocentrism-technocentrism	–	–	5.2	6.9	3.7	5.9
Environmentally significant behavior	–	–	4.5	5.9	3.2	5.0
Hofstede's theory of cultural dimensions	–	–	3.0	3.9	2.1	3.4
Maslow's hierarchy of needs	1.8	5.9	2.2	2.9	2.1	3.4
New ecological paradigm (HEP-NEP)	19.6	64.7	38.8	51.0	33.2	52.9
NIMBY	1.8	5.9	2.2	2.9	2.1	3.4
Norm activation theory	8.9	29.4	9.0	11.8	9.0	14.3
Post-materialism	5.4	17.7	12.7	16.7	10.5	16.8
Rational choice	–	–	3.0	3.9	2.1	3.4
Risk society	–	–	2.2	2.9	1.6	2.5
Self-perception theory	–	–	3.0	3.9	2.1	3.4
Social dilemmas and game theory	–	–	4.5	5.9	3.2	5.0
Social identity theory	–	–	4.5	5.9	3.2	5.0
Social learning theory	3.6	11.8	4.5	5.9	4.2	6.7
Gender socialization theory	–	–	3.0	3.9	2.1	3.4
Theory of cultural value orientations	3.6	11.8	8.2	10.8	6.8	10.9
Theory of planned behavior	3.6	11.8	20.2	26.5	15.3	24.4
Theory of reasoned action	3.6	11.8	13.4	17.7	10.5	16.8
Value orientations	1.8	5.9	14.2	18.6	10.5	16.8
Value-belief-norm	–	–	11.9	15.7	8.4	13.5
White's theory	–	–	3.0	3.9	2.1	3.4
SUM		100		100		100
Docs mentioning theor. aprch.	30.4		76.1		62.6	
Docs not mentioning theor. aprch.	69.6		23.9		37.4	
No. of docs	56	17	134	102	190	119

The second change in the discourse is related to the content of the discourse. The cut-off point sits somewhere around 1999–2000. This is the point when the theoretical diversity indicator surpassed the number of articles and did not drop below. Beyond this point, data shows greater diversity in theoretical assumptions employed for the analytical frameworks within the articles. New theories and concepts are introduced into the discourse throughout this period, which does not suggest any consolidation. Some of the most prominent theoretical developments have occurred around these turning points. For example, in 1994, Stern and Dietz coined the Value Orientation Theory; in 2000 Stern published his work defining the theory of Environmentally Significant Behavior. It was further followed by other theoretical developments, e.g., Theory of Cultural Value Orientations by Schwartz (2006).

After showing the overall trend over time, we now turn toward research the questions on the actual theories. We ask: (RQ3) Which theoretical approaches are mentioned within the theoretical frameworks of the reported empirical studies? (RQ4) How does the prevalence of different theories shift over time? Table 3 shows our results in this regard. Considering the period of 1971–2020, the Theory of Planned Behavior, HEP-NEP distinction, Theory of Reasoned Action, Postmaterialism, Value Orientations theory, Norm Activation Theory, and Value-Belief-Norm Theory are generally most visible in the academic discourse on environmental attitudes and behaviors.

Table 3 also includes columns reflecting the specific periods we identified in our analyses. Column 1 covers the initial pre-2000 period which is characterized by a few dominant theories. The New Ecological Paradigm (NEP) is mentioned in around two-thirds of all analyzed papers, that had any clear references to theoretical approaches (64.7%) followed by the Norm Activation Theory (29.4%) and Postmaterialism (17.7%). Not surprisingly, Milfont et al. (2019, p. 477) found that the two publications by Riley E. Dunlap and Kent D. Van Liere on the NEP are among the most cited publications.

Column 2 covers the period from 2000 onwards. This period of the academic discourse is marked by the rise of Value-Belief-Norm Theory, Theory of Planned Behavior, Value Orientations approach and the declining relative importance (valid % of docs) of the Norm Activation Theory, HEP-NEP theories, and Social Learning Theory. The latter theories are mentioned throughout the 2nd period, but lose some of their relative importance as other theories are mentioned as well.

**Figure 2:**
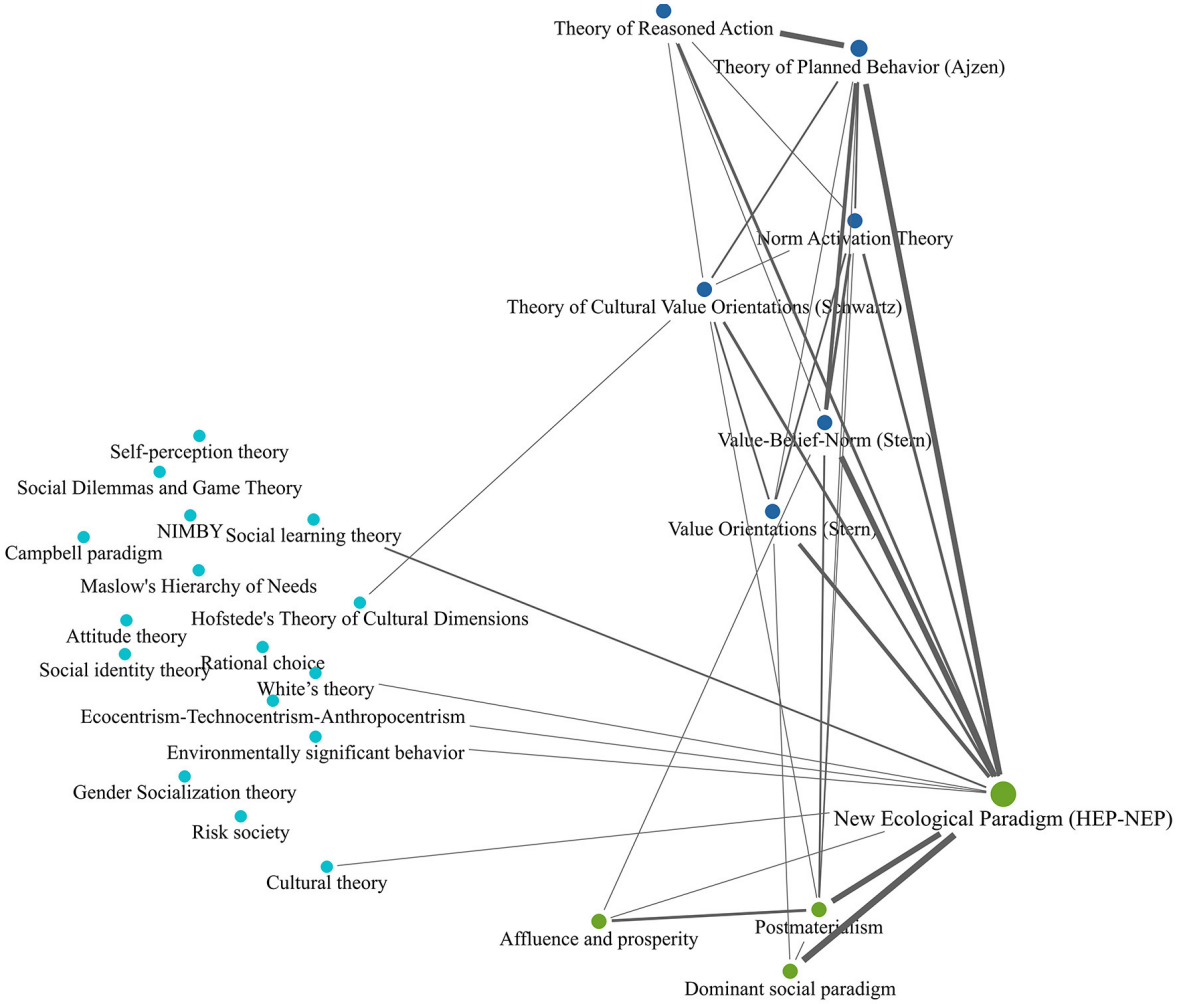
Co-occurrence of all mentioned theories in the post-2000 period. Lines represent instances of co-occurrence with a frequency ≥3, higher co-occurrence frequency means more solid line.

Considering the overall trends displayed in Figure 1 and the trajectory of specific theories (Table 3), our findings on the development of different theories and trajectories over time thus can be summarized in the following stages:

1. Up to 1993—Incipient stage of the academic discourse: initiation of the discourse and scarce publications with empirical material throughout the period.
2. 1993–2000—Ignition stage: (1) increased but fluctuating intensity (continuous line in the Figure 1) as measured by articles per year; (2) moving average timeline goes up; (3) introduction of theoretical richness (broken line in Figure 1 goes up and stays high).
3. Post 2000—Pronounced discourse, i.e., relatively intense and theoretically diverse period: (1) diversity does not drop lower than the total amount of articles (broken line in Figure 1); (2) intensity stays relatively high despite fluctuations (continuous line in Figure 1).

### 4.2. The co-occurrence of theories

The second part of the results section focuses on the relations between the different theories and addresses the research question: (RQ5) How often do the theoretical assumptions co-occur in academic texts? And (RQ6) which ones do co-occur most often? Addressing these questions is important given that the theoretical frameworks presented in the articles are usually comprised of several theoretical approaches and concepts. If we count the total number of segments coded by any theoretical code (mentioning a theoretical assumption), we get a total of 404 tokens. Throughout the analysis, we identified 26 repeated theoretical assumptions (mentioned at least three times) and 108 instances of mentioning other theoretical assumptions (a total of 134 types of theories). The type-token ratio^3^ is 33.2%. This indicates relatively high theoretical variation in the academic discourse.

**Figure 3:**
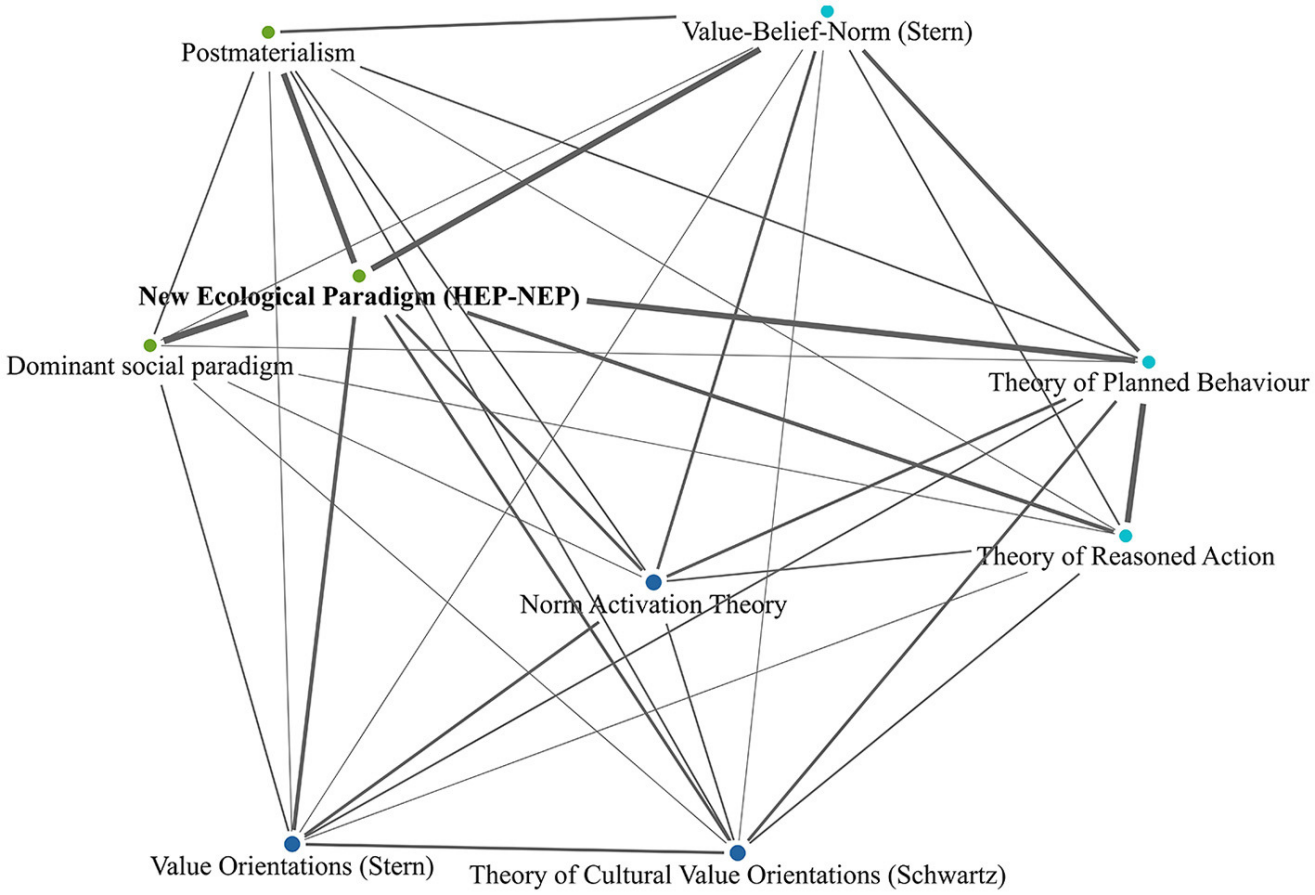
Co-occurrence of most frequently mentioned theories in the post-2000 articles (mentioned ≥13 times). Lines represent instances of co-occurrence with a frequency ≥3, higher co-occurrence frequency means more solid line.

To display the connection between the different theories and concepts, we applied a code co-occurrence analysis. This analysis helps us to identify the semantic networks formed within the theoretical frameworks. Given the uptick in the number of theories in the post-2000 period, the results presented in Figures 2, 3 are limited to this period. Further, Figure 2 displays the results for all theories, whereas Figure 3 is limited to the most frequent theories, that is theories mentioned at least 13 times throughout the period.

On the right side of the co-occurrence map of all theories (displayed in Figure 2), we see HEP-NEP as the most prominent theories employed in the academic discourse on environmental attitudes and behavior. The theories providing micro-level explanations are positioned in the top-center of the map. Macro-theories explaining group or country-level developments tend to be positioned toward the bottom-center of the map. We can see the group of less prominent and less co-occurring theories on the left side of Figure 2.

To analyze the more recent and persistent collaborations, we further look at the most frequently mentioned theories (see Figure 3) in the post-2000 period. The theoretical assumptions within the co-occurrence map can be classified into persistent collaborators, frequent collaborators, and dependents. Persistent collaborators are rarely mentioned alone in the articles. They usually lead to the mention of other theoretical approaches and therefore often co-exist with them. These are kinds of collaborators that activate the use of other theoretical approaches. Frequent collaborators are many times mentioned alone but also are frequently engaging and co-occur with other theoretical approaches. Whereas, dependents refer to theoretical approaches that seldom or never would be mentioned in the articles unless their collaborator theories. Dependents are only mentioned if their collaborators are mentioned.

Again, we see the HEP-NEP theoretical approaches as the most frequently referred approaches in the discourse. It also serves as a persistent collaborator. 52.9% of all documents that mention theoretical approaches, refer to HEP-NEP (see Table 3). The Dominant Social Paradigm precedes HEP-NEP, but is a dependent element in this discourse, as Dominant Social Paradigm is mentioned only if HEP or NEP is also mentioned. Postmaterialism and Value-Belief-Norm theories are strongly associated with HEP-NEP, but are not completely dependent on HEP-NEP (co-occurs with HEP-NEP 13 times out 20 mentions and 11 times out of 16 mentions accordingly). The Theory of Planned Behavior is the second strongest persistent collaborator (24.4% of all documents that mention theoretical approaches, refer to it, see Table 3). The theory of Reasoned Action is often (13 times out of 20) mentioned as preceding the Theory of Planned Behavior. However, it is treated as a frequent collaborator of the Theory of Planned Behavior, but is not a dependent.

All the theories portrayed in the map, except for Dominant Social Paradigm and the two persistent collaborators, serve as frequent collaborators with other theories, i.e., are frequently mentioned together with other theories. These findings elaborate on the dominance of the HEP-NEP approaches and Theory of Planned Behavior theories. They are not only the most frequently mentioned, but they also are the ones that activate the mentioning of several other theories, that would not appear in the discourse if not the HEP-NEP approaches and the Theory of Planned Behavior.

### 4.3. Empirical background/evidence brought forward in the texts

The empirical support brought forward in the academic discourse allows assessing the robustness of the cumulative knowledge in the field. We thus ask (RQ7) what is the geographical coverage of the reported empirical studies, (RQ8) does the sampling cover entire societies or only some social groups, (RQ9) what is the chronology and recentness of the empirical studies, and (RQ10) which explanatory variables are used in the empirical studies.

We start with the recentness of the empirical studies. Fieldwork dates were reported only in 108 different studies mentioned in 97 documents (out of 190 documents). The average delay between an empirical study and a publication was 5 years. There is a slight difference between the group of publications published before 2000 and the ones published after 2000—the average in the former is 4 years and 5 years in the latter. The delay thus increased over time.

As for the geographical coverage, Oreg and Katz-Gerro (2006) have noticed that a huge percentage of studies use local, single-country, and small samples when analyzing predictors of pro-environmental behavior. Our analysis supports their finding. After interrogating the sample of articles, we also find limited geographical coverage of the empirical studies on environmental attitudes and behaviors (see Table 4). Empirical studies from the United States dominate the empirical evidence base. 50.5% of all documents report a study from, on, or including the United States. Many countries are mentioned once or twice due to their participation in international research programs such as International Social Survey Program (ISSP) or World Values Survey (WVS).

**Table 4:** Geographical coverage of the reported empirical studies.

**Countries**	**No. of documents mentioning a study in the country**
United States	101
Canada	25
United Kingdom	19
Germany	17
Spain	15
Netherlands	13
Italy	12
China, Switzerland	11
Sweden, Norway	10
Australia, New Zealand, Turkey	9
Bulgaria, Japan, Mexico, Slovenia	8
Austria, Argentina, Czech Republic, Chile, Ireland, Israel, Poland	6
Finland, Philippines, Portugal, Russian Federation, South Africa	5
Denmark, France, Latvia, Peru	4
Belgium, Hungary, India, Lithuania, Republic of Korea, Romania, Trinidad and Tobago, Ukraine	3
Brazil, Croatia, Dominican Republic, Estonia, Ethiopia, Indonesia, Moldova, Singapore, Slovakia, Thailand, Vietnam	2
Albania, Bangladesh, Belarus, Burkina Faso, Costa Rica, Cyprus, Cuba, Ecuador, Egypt, El Salvador, Georgia, Ghana, Greece, Iceland, Jordan, Malaysia, Mali, Morocco, North Macedonia, Pakistan, Panama, Paraguay, Rwanda, Taiwan, Uganda, Uruguay, Venezuela, Zambia, Zimbabwe	1
Documents with country codes	191
Documents without country codes	9
Analyzed documents, Total	200

Our results are in line with analyses and statements regarding diverse environmental issues. Fairbrother et al. (2019) also note that the United States dominates the articles on attitudes toward climate change. McCright et al. (2016) conclude that there is a risk of over-generalizing from the experience of that one exceptional country. Similar, Fairbrother (2016, p. 361) observed that in the literature on public support for environmental action, “a disproportionate number of studies have employed U.S. data” and Milfont et al. (2019, p. 476) in their analysis of EB articles state that North Americans authored almost 70% of all the papers ever published in the journal. Even a more recent study (Zhu et al., 2021) has found that the United States is still the leading country in the field, followed by other English-speaking countries.

Our results and the observations of these other scholars indicate that the field of studies on environmental attitudes and behaviors heavily focuses on the North American context, which can be problematic when assuming the global applicability of any theory. Further, alongside these geographical concerns, theories that claim a higher level of universality also ought to be tested on diverse social groups. Ideally, the empirical evidence should include societies in their entirety as it allows for a broad validation—which might not be given if only some specific social groups are included. Table 5 presents our results in this regard. It shows a strong dependency on data coming from narrow social backgrounds.

**Table 5:** Data sources, populations and samples of the reported empirical studies.

	**Categories**	**Documents**	**Percentage**
Data sources	Original studies	163	81.5
	Regular national or regional studies	23	11.5
	International/ multinational studies	10	5.0
	Documents with codes	193	96.5
Populations	Social group or organization	101	50.5
	Territory within country	63	31.5
	Country	25	12.5
	Multiple countries	10	5.0
	Documents with codes	193	96.5
Samples	Non-representative	128	64.0
	Representative	69	34.5
	Documents with codes	192	96.0

Most of the empirical studies are conducted with specific social groups or in territories within countries. 62.6% of all analyzed articles relied on non-representative samples. Further analysis of the co-occurrence of theory codes with “Representativeness” codes showed that representative samples are more often reported in articles mentioning New Ecological Paradigm, Postmaterialism, and Theory of Planned Behavior. 81.1% of all analyzed documents drew on empirical studies originally conducted by the authors themselves.

Code configuration analysis showed that the empirical evidence is most frequently based on original non-representative studies with various social groups or territories within countries. Representative samples, country and multi-country studies are under-represented, which undermines the further development of more robust validation of theories.

Our final research question considers the types of explanatory (independent) variables employed in the reported empirical studies. Results show that the majority of studies focus on socio-psychological (individual) level constructs and behavioral variables (151 or 79.5% of all documents). These socio-psychological and behavioral variables included: knowledge, attitudes, perceptions, beliefs, concern, awareness, assessment, ideology, worldview, behavior, norm, intention, preferences, values, feelings, motivation, opinions, willingness, political ideology, satisfaction, trust, belonging, connectedness, empathy, experience, preferences, etc. (inductively generated list). More than half of all documents have employed socio-structural (positional) variables (107 or 56.3% of all documents). Positional variables included age, gender, occupation, residence, ethnicity, education, income, household composition, and other measures.

Considering the two periods, we can also report that the academic field has witnessed a slight shift toward a more frequent use of socio-psychological and behavioral variables in the explanatory models. As displayed in Table 6, socio-psychological and behavioral variables are more often employed in the post-2000 period. Socio-structural variables have been more often employed in the pre-2000 period. Our finding, that psychology-related concepts are most often used in the articles, is in line with the Milfont et al. (2019, p. 490) finding from co-citation analysis, that psychology-related journals are most often cited within EB, and highlights the dominance of psychology in research on environmental attitudes and behaviors.

**Table 6:** Explanatory variables used in the analyzed articles (percentages of documents mentioning the variables).

**Groups of explanatory variables**	**Pre-2000**	**Post-2000**
Socio-psychological (individual) constructs and behaviors	69.6	83.3
Socio-structural (positional) variables	60.7	52.1
Situational (contextual) variables	21.4	22.2
Macro variables	8.9	9.7
Documents with codes	82.1	92.4
Documents without codes	17.9	7.6
Analyzed documents	56	144

## 5. Conclusion and discussion

Our analysis aimed to investigate systematically the order and content of the academic discourse on environmental attitudes and behaviors. We thus conducted a systematic literature review and content analysis of all articles using empirical evidence published in the journal “Environment and Behavior” (EB) from 1971 to 2020. EB was chosen as a typical case based on a systematic analysis of all SCOPUS and Web of Science entries and also as a rich case with proven scientific impact in the field (Milfont et al., 2019; Zhu et al., 2021).

As pointed out in the background section, the development of new academic knowledge and the attention given to different topics can be seen as cyclical processes. The shift to new paradigms is preceded by a phase of competing views (Kuhn, 2012) and academic discourse is also subject to issue attention cycles similar to the media discourse (Downs, 1972). Our analysis shows that the academic discourse on environmental attitudes and behaviors has indeed undergone several development stages: an incipient stage up to 1993, an ignition stage in the period between 1993 and 2000, and a pronounced discourse stage in the post-2000 period. The post-2000 period has witnessed a persistent intensity and growth of theoretical diversity but has not yet brought the discourse to a consolidation (or fatigue) stage.

There were five attention peaks with an average of 2.75-year attention span in the post-2000 period. This shows that the academic discourse is still hectic, and a knowledge plateau has not yet developed, i.e., in Kuhn's (2012 terms, no dominant scientific paradigm has been established in the field yet. The Theory of Planned Behavior, Human Exemptionalist and New Ecological Paradigms), Theory of Reasoned Action, Postmaterialism, Value Orientations theory, Norm Activation Theory, and Value-Belief-Norm Theory are generally the most visible in the academic discourse on environmental attitudes and behaviors. Human Exemptionalist and New Ecological Paradigms constitute the most prominent theoretical references employed in the academic discourse. Together with the Theory of Planned Behavior, they serve as persistent collaborators, i.e., co-occur frequently with other theories within the theoretical frameworks of the analyzed articles. Yet, it has to be noted, that theoretical frameworks often bear instrumental references to previously published research without any detailed discussion of the applied theories, theses, and paradigms.

The majority of studies have focused on socio-psychological (individual) level constructs and behavioral variables. The academic field has witnessed a slight shift toward more frequent use of these variables in the post-2000 period (with socio-structural/positional variables being more prominent in the pre-2000 period). Situational and macro-level variables are underemployed. This also points to the domination of socio-psychological approaches over sociological, economic, or other approaches (that would more often deal with larger social systems instead of individual-level characteristics). Theories that deal with questions at the level of individuals and small groups are most frequently employed in exploring environmental attitudes and behavior. As Guagnano et al. (1995) have pointed out, socio-psychological variables constitute only part of the story, and contextual variables need to be better accounted for in explanatory models (the suggested model was titled the A-B-C model). Contextualizing the empirically proven patterns of individual environmental attitudes and behaviors within broader explanatory frameworks, usually provided by general and macro theories, should be further sought.

We also assessed the strength of the empirical evidence brought forward in support of the considered theories. It can first be noted, that our findings go hand in hand with the conclusion of Arbuckle et al. (2015, p. 210), who argued that synthesizing results and making more general interpretations is difficult because of the diversity of measures used in the empirical studies. Our results show that studies most frequently are based on original non-representative studies of limited social groups or territories within countries. Representative samples, country-wide studies, and multi-country studies are under-represented. Furthermore, a lack of studies that use data from non-Western countries hinders a discussion of the global applicability of the different theories, as feedback from these countries is rare. In short, the empirical evidence for environmental attitudes and behaviors has:

- cross-cultural validation issues: evidence comes from a limited set of cultures/countries, mostly from North America;
- recentness issues: it takes 5 years on average until the data is published;
- limited demographic variation and socio-structural universality issues: evidence is most often drawn from social groups or territories rather than larger national or cross-national samples; data from cross-cultural surveys, e.g., WVS or ISSP are underemployed.

These limitations result in a lack of universality of the findings—results cannot be generalized nor be considered directly applicable to other locations and cultures. To ascertain the cross-cultural applicability of theories, the geographical coverage of the empirical studies needs to be widened. At the same time, secondary data analysis and the reuse of available survey data is underemployed. Reuse of data for secondary analysis is greatly recommended, especially when the data covers diverse social systems. If the item structure fits the research questions, it is better to conduct secondary data analysis than to conduct an original study of small and/or non-representative samples. Our detailed analysis, however, was limited to the journal Environment and Behavior.

Future research needs to expand our analysis to other journals to see if these biases are specific or also visible in other outlets. It would be also interesting to see if these theories also influenced scholars in other areas of research such as conservation social scientists, climate researchers, or sustainability research. It would be useful to utilize computer-based content analyses and the growing AI-based methods to extend the scope and the scale of the present analysis.

Further research could explore why (or to what extent) the field of research on environmental attitudes and behaviors is less receptive of non-Northern-American scholars and epistemologies, what is the broader implications of the overrepresentation of US and other English-speaking countries in the evidence-body of the field. We see a broader trend in the academic world, which our results also tap into. Taking an extremely critical position, some researchers speak of epistemological hegemony (de Sousa Santos, 2015; Noda, 2020). The post-colonial theory could be further employed to study the implications of these trends for knowledge development and future prospects of the field.

## Data availability statement

The raw data supporting the conclusions of this article will be made available by the authors, without undue reservation.

## Author contributions

AT and MH have both contributed to the conception and design of the study. AT implemented the systematic literature review, organized the database, coded the texts, conducted the quantitative content analysis with MAXQDA, and wrote the first draft of the manuscript. MH contributed to the development of search strings and implementation of the screening steps as reported in the PRISMA Flow Diagram. He also contributed to the development of coding schemes and coding of the articles, wrote sections of the manuscript related to the background of the study, and the discussion of the results. Both authors contributed to the article, and manuscript revision, and have read and approved the submitted version.

## Figures and Tables

**Figure A1:**
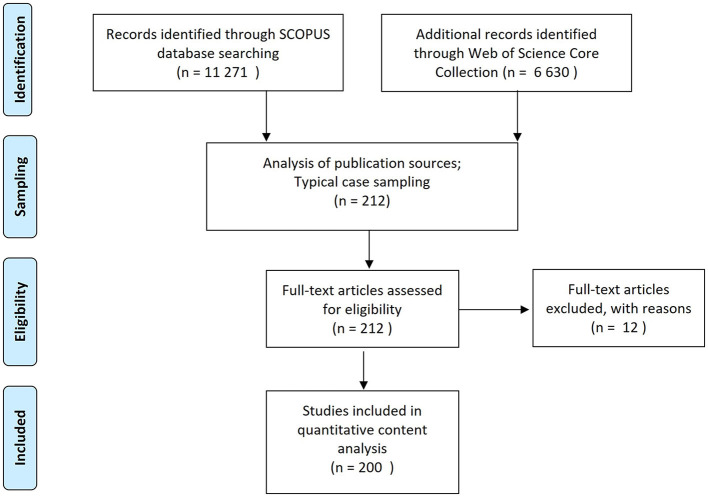
PRISMA flow diagram.

## Footnotes

^1^There were 6,630 records identified through Web of Science Core Collection database, using search string: TOPIC: (“environmental attitudes”) OR TOPIC: (environmental^*^ NEAR/3 behav^*^); Refined by: DOCUMENT TYPES: (ARTICLE OR BOOK CHAPTER) AND WEB OF SCIENCE INDEX: (WOS.BHCI OR WOS.SSCI OR WOS.ESCI); Timespan: All years; Indexes: SCI-EXPANDED, SSCI, A&HCI, CPCI-S, CPCI-SSH, ESCI”. And there were 11,271 records identified through SCOPUS database, using search string: [TITLE-ABS-KEY (environmental AND attitudes) OR TITLE-ABS-KEY (environmental^*^ AND near 3 behav^*^)] AND [LIMIT-TO (SUBJAREA, “ENVI”) OR LIMIT-TO (SUBJAREA, “SOCI”) OR LIMIT-TO (SUBJAREA, “MULT”) AND (LIMIT-TO (DOCTYPE, “ar”) OR LIMIT-TO (DOCTYPE, “ch”) OR LIMIT-TO (DOCTYPE, “bk”)].

^2^Based on this pilot-coding-exercise, coding ambiguities, uncertainties, and incompleteness was discussed between the authors and with a group of independent researchers. For measuring inter-coder agreement, 31 texts have been assigned for project-independent coders. Agreement between coding completed by project researcher (Coder 1) and the project-independent researchers (Coder 2 and Coder 3) was measured using several measurements. Code occurrence in the document (Total) reached the inter-coder agreement of 93.16% (compared to texts coded by Coder 2) and 95.66% (compared to texts coded by Coder 3). Code frequency in the document (Total) turned 91.32 and 93.95% agreement levels. Two code families resulted in higher disagreements: “status of the theory” code family was removed from further analysis (highly subjective) and “Representativeness” coding was adjusted.

^3^Type-token ratio usually measures the vocabulary variation within a text corpus. In this case it measures the variation of theoretical approaches within the studied articles. It shows if only the same theories are being used throughout all academic texts, or a variety of different approaches is used. One hundred percent would mean that every text mentions different theoretical approach. Zero percent would mean that a single theory is being used in all the academic texts.

## Appendix 1. Systematic review protocol and PRISMA flow diagram

Several pilot-sampling-exercises were completed before deciding on the final sampling strategy. The first pilot-sampling-exercise included searching for keywords “environmental attitudes,” “environmental concern” and “environmental behav*.” The databases' search engines returned tens of thousands of entries with less than half of those relevant for our research. A closer reading of titles proved that search should be limited to articles, books and book chapters, and that it can also be limited to some subject areas: environmental sciences, social sciences and multidisciplinary studies. Excluding “concern” as keyword and limiting the search by the type of publications and by the subject areas improved the share of relevant texts. The second pilot-sampling-exercise included search terms related to the main theories in the field, e.g., “postmaterialism,” “theory of planned behavior,” etc. This indicated the need to use lemmas and to use “near” function in the search strings (e.g., environmental behavior vs. environmentally significant behavior).
