# Supplementary material for: Dynamics and landscape of academic discourse on environmental attitudes and behaviors since the 1970s
Source: Front Sociol. 2023 Jul 14;8:1136972. doi: 10.3389/fsoc.2023.1136972 (PMC10382132; doi:10.3389/fsoc.2023.1136972)
Supplement: Supplementary file 1 [file Data_Sheet_1.DOCX]

Supplementary Material

Dynamics and Landscape of Academic Discourse on Environmental Attitudes and Behaviors since the 1970s

**Audrone Telesiene^1*^, Markus Hadler^2, 3^**

^1^ Civil Society and Sustainability Research Group, Kaunas University of Technology, Kaunas, Lithuania

^2^ Department of Sociology, University of Graz, Graz, Austria

^3^ Department of Sociology, Macquarie University, Sydney, Australia

*** Correspondence:**Audrone Telesiene
[audrone.telesiene@ktu.lt](mailto:audrone.telesiene@ktu.lt)

# Supplementary Data

This sheet contains a list of the studies included in the quantitative content analysis (n=200), the results of which are presented in the paper.

# Studies included in quantitative content analysis (n=200)

Adeola, F.O. (1994) ‘Environmental Hazards, Health, and Racial Inequity in Hazardous Waste Distribution’, Environment and Behavior, 26(1), pp. 99–126. Available at: https://doi.org/10.1177/0013916594261006.

Adeola, F.O. (2000) ‘Endangered Community, Enduring People: Toxic Contamination, Health, and Adaptive Responses in a Local Context’, Environment and Behavior, 32(2), pp. 209–249. Available at: https://doi.org/10.1177/00139160021972504.

Allen, J.B. and Ferrand, J.L. (1999) ‘Environmental Locus of Control, Sympathy, and Proenvironmental Behavior: A Test of Geller’s Actively Caring Hypothesis’, Environment and Behavior, 31(3), pp. 338–353. Available at: https://doi.org/10.1177/00139169921972137.

Arbuckle, J.G., Morton, L.W. and Hobbs, J. (2015) ‘Understanding Farmer Perspectives on Climate Change Adaptation and Mitigation: The Roles of Trust in Sources of Climate Information, Climate Change Beliefs, and Perceived Risk’, Environment and Behavior, 47(2), pp. 205–234. Available at: https://doi.org/10.1177/0013916513503832.

Arbuthnot, J. (1977) ‘The Roles of Attitudinal and Personality Variables in the Prediction of Environmental Behavior and Knowledge’, Environment and Behavior, 9(2), pp. 217–232. Available at: https://doi.org/10.1177/001391657792004.

Arp, W. and Kenny, C. (1996) ‘Black Environmentalism in the Local Community Context’, Environment and Behavior, 28(3), pp. 267–282. Available at: https://doi.org/10.1177/0013916596283001.

Audirac, I. and Shoemyen, A.H. (1989) ‘The Use of Converse’s Mass Belief Systems Model to Measure Public Opinion on Land Use Regulation: A Comment on deHaven-Smith’s Article’, Environment and Behavior, 21(5), pp. 620–629. Available at: https://doi.org/10.1177/0013916589215006.

Aytülkasapoğlu, M. and Ecevit, M.C. (2002) ‘Attitudes and Behavior toward the Environment: The Case of Lake Burdur in Turkey’, Environment and Behavior, 34(3), pp. 363–377. Available at: https://doi.org/10.1177/0013916502034003005.

Bailey, J.O. et al. (2015) ‘The Impact of Vivid Messages on Reducing Energy Consumption Related to Hot Water Use’, Environment and Behavior, 47(5), pp. 570–592. Available at: https://doi.org/10.1177/0013916514551604.

Barr, S. (2007) ‘Factors Influencing Environmental Attitudes and Behaviors: A U.K. Case Study of Household Waste Management’, Environment and Behavior, 39(4), pp. 435–473. Available at: https://doi.org/10.1177/0013916505283421.

Benjamin, D., Por, H.-H. and Budescu, D. (2017) ‘Climate Change Versus Global Warming: Who Is Susceptible to the Framing of Climate Change?’, Environment and Behavior, 49(7), pp. 745–770. Available at: https://doi.org/10.1177/0013916516664382.

Berenguer, J. (2007) ‘The Effect of Empathy in Proenvironmental Attitudes and Behaviors’, Environment and Behavior, 39(2), pp. 269–283. Available at: https://doi.org/10.1177/0013916506292937.

Berger, I.E. (1997) ‘The Demographics of Recycling and the Structure of Environmental Behavior’, Environment and Behavior, 29(4), pp. 515–531. Available at: https://doi.org/10.1177/001391659702900404.

Blake, D.E. (2001) ‘Contextual Effects on Environmental Attitudes and Behavior’, Environment and Behavior, 33(5), pp. 708–725. Available at: https://doi.org/10.1177/00139160121973205.

Blamey, R. (1998) ‘The Activation of Environmental Norms: Extending Schwartz’s Model’, Environment and Behavior, 30(5), pp. 676–708. Available at: https://doi.org/10.1177/001391659803000505.

Bodur, M. and Sarigöllü, E. (2005) ‘Environmental Sensitivity in a Developing Country: Consumer Classification and Implications’, Environment and Behavior, 37(4), pp. 487–510. Available at: https://doi.org/10.1177/0013916504269666.

Bonnes, M., Passafaro, P. and Carrus, G. (2011) ‘The Ambivalence of Attitudes Toward Urban Green Areas: Between Proenvironmental Worldviews and Daily Residential Experience’, Environment and Behavior, 43(2), pp. 207–232. Available at: https://doi.org/10.1177/0013916509354699.

Botetzagias, I. and van Schuur, W. (2012) ‘Active Greens: An Analysis of the Determinants of Green Party Members’ Activism in Environmental Movements’, Environment and Behavior, 44(4), pp. 509–544. Available at: https://doi.org/10.1177/0013916510393278.

Brick, C. and Lewis, G.J. (2016) ‘Unearthing the “Green” Personality: Core Traits Predict Environmentally Friendly Behavior’, Environment and Behavior, 48(5), pp. 635–658. Available at: https://doi.org/10.1177/0013916514554695.

Brody, S.D., Highfield, W. and Alston, L. (2004) ‘Does Location Matter?: Measuring Environmental Perceptions of Creeks in Two San Antonio Watersheds’, Environment and Behavior, 36(2), pp. 229–250. Available at: https://doi.org/10.1177/0013916503256900.

Brown, B.B. and Werner, C.M. (1985) ‘Social Cohesiveness, Territoriality, and Holiday Decorations: The Influence of Cul-de-Sacs’, Environment and Behavior, 17(5), pp. 539–565. Available at: https://doi.org/10.1177/0013916585175001.

Brown, R.S., Williams, C.W. and Lees-Haley, P.R. (1993) ‘The Effect of Hindsight Bias on Fear of Future Illness’, Environment and Behavior, 25(4), pp. 577–585. Available at: https://doi.org/10.1177/0013916593254002.

Bruvold, W.H. (1973) ‘Belief and Behavior as Determinants of Environmental Attitudes’, Environment and Behavior, 5(2), pp. 202–218. Available at: https://doi.org/10.1177/001391657300500205.

Burger, J. et al. (1998) ‘Gender Differences in Recreational Use, Environmental Attitudes, and Perceptions of Future Land Use at the Savannah River Site’, Environment and Behavior, 30(4), pp. 472–486. Available at: https://doi.org/10.1177/001391659803000403.

Buttel, F.M. and Flinn, W.L. (1978) ‘The Politics of Environmental Concern: The Impacts of Party Identification and Political Ideology on Environmental Attitudes’, Environment and Behavior, 10(1), pp. 17–36. Available at: https://doi.org/10.1177/0013916578101002.

Byrka, K., Kaiser, F.G. and Olko, J. (2017) ‘Understanding the Acceptance of Nature-Preservation-Related Restrictions as the Result of the Compensatory Effects of Environmental Attitude and Behavioral Costs’, Environment and Behavior, 49(5), pp. 487–508. Available at: https://doi.org/10.1177/0013916516653638.

Cao, X., Mokhtarian, P.L. and Handy, S.L. (2009) ‘No Particular Place to Go: An Empirical Analysis of Travel for the Sake of Travel’, Environment and Behavior, 41(2), pp. 233–257. Available at: https://doi.org/10.1177/0013916507310318.

Carrus, G., Bonaiuto, M. and Bonnes, M. (2005) ‘Environmental Concern, Regional Identity, and Support for Protected Areas in Italy’, Environment and Behavior, 37(2), pp. 237–257. Available at: https://doi.org/10.1177/0013916504269644.

Cheng, J.C.-H. and Monroe, M.C. (2012) ‘Connection to Nature: Children’s Affective Attitude Toward Nature’, Environment and Behavior, 44(1), pp. 31–49. Available at: https://doi.org/10.1177/0013916510385082.

Cini, F., Leone, L. and Passafaro, P. (2012) ‘Promoting Ecotourism Among Young People: A Segmentation Strategy’, Environment and Behavior, 44(1), pp. 87–106. Available at: https://doi.org/10.1177/0013916510389313.

Clarke, A., Bell, P.A. and Peterson, G.L. (1999) ‘The Influence of Attitude Priming and Social Responsibility on the Valuation of Environmental Public Goods Using Paired Comparisons’, Environment and Behavior, 31(6), pp. 838–857. Available at: https://doi.org/10.1177/00139169921972371.

Clayton, S. (1998) ‘Preference for Macrojustice Versus Microjustice in Environmental Decisions’, Environment and Behavior, 30(2), pp. 162–183. Available at: https://doi.org/10.1177/0013916598302003.

Collado, S. and Corraliza, J.A. (2015) ‘Children’s Restorative Experiences and Self-Reported Environmental Behaviors’, Environment and Behavior, 47(1), pp. 38–56. Available at: https://doi.org/10.1177/0013916513492417.

Corraliza, J.A. and Berenguer, J. (2000) ‘Environmental Values, Beliefs, and Actions: A Situational Approach’, Environment and Behavior, 32(6), pp. 832–848. Available at: https://doi.org/10.1177/00139160021972829.

Corral-Verdugo, V. (2002) ‘A Structural Model of Proenvironmental Competency’, Environment and Behavior, 34(4), pp. 531–549. Available at: https://doi.org/10.1177/00116502034004008.

Costantini, E. and Hanf, K. (1972) ‘Environmental Concern and Lake Tahoe: A Study of Elite Perceptions, Backgrounds, and Attitudes’, Environment and Behavior, 4(2), pp. 209–242. Available at: https://doi.org/10.1177/001391657200400204.

Cottrrel, S.P. (2003) ‘Influence of sociodemographics and environmental attitudes on general responsible environmental behavior among recreational boaters’, Environment and Behavior, 35(3), pp. 347–375. Available at: https://doi.org/10.1177/0013916503035003003.

Cranz, G. et al. (2014) ‘Communicating Sustainability: A Postoccupancy Evaluation of the David Brower Center’, Environment and Behavior, 46(7), pp. 826–847. Available at: https://doi.org/10.1177/0013916513475449.

Daamen, D.D.L. et al. (2001) ‘Improving Environmental Behavior in Companies: The Effectiveness of Tailored Versus Nontailored Interventions’, Environment and Behavior, 33(2), pp. 229–248. Available at: https://doi.org/10.1177/00139160121972963.

Deng, J., Walker, G.J. and Swinnerton, G. (2006) ‘A Comparison of Environmental Values and Attitudes Between Chinese in Canada and Anglo-Canadians’, Environment and Behavior, 38(1), pp. 22–47. Available at: https://doi.org/10.1177/0013916505278458.

Diekmann, A. and Franzen, A. (1999) ‘The Wealth of Nations and Environmental Concern’, Environment and Behavior, 31(4), pp. 540–549. Available at: https://doi.org/10.1177/00139169921972227.

Dietz, T., Stern, P.C. and Guagnano, G.A. (1998) ‘Social Structural and Social Psychological Bases of Environmental Concern’, Environment and Behavior, 30(4), pp. 450–471. Available at: https://doi.org/10.1177/001391659803000402.

Dixon, G.N. et al. (2015) ‘Using Comparative Feedback to Influence Workplace Energy Conservation: A Case Study of a University Campaign’, Environment and Behavior, 47(6), pp. 667–693. Available at: https://doi.org/10.1177/0013916513520417.

Do Valle, P.O. et al. (2004) ‘Behavioral Determinants of Household Recycling Participation: The Portuguese Case’, Environment and Behavior, 36(4), pp. 505–540. Available at: https://doi.org/10.1177/0013916503260892.

Do Valle, P.O.D. et al. (2005) ‘Combining Behavioral Theories to Predict Recycling Involvement’, Environment and Behavior, 37(3), pp. 364–396. Available at: https://doi.org/10.1177/0013916504272563.

Duffy, S. and Verges, M. (2010) ‘Forces of Nature Affect Implicit Connections With Nature’, Environment and Behavior, 42(6), pp. 723–739. Available at: https://doi.org/10.1177/0013916509338552.

Dunlap, R.E. (1975) ‘The Impact of Political Orientation On Environmental Attitudes and Actions’, Environment and Behavior, 7(4), pp. 428–454. Available at: https://doi.org/10.1177/001391657500700402.

Duvall, J. (2013) ‘Using Engagement-Based Strategies to Alter Perceptions of the Walking Environment’, Environment and Behavior, 45(3), pp. 303–322. Available at: https://doi.org/10.1177/0013916511423808.

Ebreo, A., Hershey, J. and Vining, J. (1999) ‘Reducing Solid Waste: Linking Recycling to Environmentally Responsible Consumerism’, Environment and Behavior, 31(1), pp. 107–135. Available at: https://doi.org/10.1177/00139169921972029.

Ecklund, E.H. et al. (2017) ‘Examining Links Between Religion, Evolution Views, and Climate Change Skepticism’, Environment and Behavior, 49(9), pp. 985–1006. Available at: https://doi.org/10.1177/0013916516674246.

Edgerton, E., McKechnie, J. and Dunleavy, K. (2009) ‘Behavioral Determinants of Household Participation in a Home Composting Scheme’, Environment and Behavior, 41(2), pp. 151–169. Available at: https://doi.org/10.1177/0013916507311900.

Evans, G.W. et al. (2007) ‘Young Children’s Environmental Attitudes and Behaviors’, Environment and Behavior, 39(5), pp. 635–658. Available at: https://doi.org/10.1177/0013916506294252.

Ewert, A. and Baker, D. (2001) ‘Standing for Where You Sit: An Exploratory Analysis of the Relationship between Academic Major and Environment Beliefs’, Environment and Behavior, 33(5), pp. 687–707. Available at: https://doi.org/10.1177/00139160121973197.

Ewing, G. (2001) ‘Altruistic, Egoistic, and Normative Effects on Curbside Recycling’, Environment and Behavior, 33(6), pp. 733–764. Available at: https://doi.org/10.1177/00139160121973223.

Farrer, B. (2016) ‘An Experiment Assessing How Different Forms of Utility Inform the Choices of Environmental Activists’, Environment and Behavior, 48(7), pp. 885–904. Available at: https://doi.org/10.1177/0013916515572696.

Fritsche, I. and Häfner, K. (2012) ‘The Malicious Effects of Existential Threat on Motivation to Protect the Natural Environment and the Role of Environmental Identity as a Moderator’, Environment and Behavior, 44(4), pp. 570–590. Available at: https://doi.org/10.1177/0013916510397759.

Gamba, R.J. and Oskamp, S. (1994) ‘Factors Influencing Community Residents’ Participation in Commingled Curbside Recycling Programs’, Environment and Behavior, 26(5), pp. 587–612. Available at: https://doi.org/10.1177/0013916594265001.

Gatersleben, B. et al. (2019) ‘Moral, Wasteful, Frugal, or Thrifty? Identifying Consumer Identities to Understand and Manage Pro-Environmental Behavior’, Environment and Behavior, 51(1), pp. 24–49. Available at: https://doi.org/10.1177/0013916517733782.

Gatersleben, B., Steg, L. and Vlek, C. (2002) ‘Measurement and Determinants of Environmentally Significant Consumer Behavior’, Environment and Behavior, 34(3), pp. 335–362. Available at: https://doi.org/10.1177/0013916502034003004.

Geiger, S.M. et al. (2020) ‘Meditating for the Planet: Effects of a Mindfulness-Based Intervention on Sustainable Consumption Behaviors’, Environment and Behavior, 52(9), pp. 1012–1042. Available at: https://doi.org/10.1177/0013916519880897.

Gelissen, J. (2007) ‘Explaining Popular Support for Environmental Protection: A Multilevel Analysis of 50 Nations’, Environment and Behavior, 39(3), pp. 392–415. Available at: https://doi.org/10.1177/0013916506292014.

Geller, E.S. (1995) ‘Actively Caring for the Environment: An Integration of Behaviorism and Humanism’, Environment and Behavior, 27(2), pp. 184–195. Available at: https://doi.org/10.1177/0013916595272004.

Gökşen, F., Adaman, F. and Zenginobuz, E.Ü. (2002) ‘On Environmental Concern, Willingness to Pay, and Postmaterialist Values: Evidence from Istanbul’, Environment and Behavior, 34(5), pp. 616–633. Available at: https://doi.org/10.1177/0013916502034005003.

Gooch, G.D. (1995) ‘Environmental Beliefs and Attitudes in Sweden and the Baltic States’, Environment and Behavior, 27(4), pp. 513–539. Available at: https://doi.org/10.1177/0013916595274004.

Groshong, L. et al. (2020) ‘Attitudes About Perceived Park Safety Among Residents in Low-Income and High Minority Kansas City, Missouri, Neighborhoods’, Environment and Behavior, 52(6), pp. 639–665. Available at: https://doi.org/10.1177/0013916518814291.

Gu, D. et al. (2020) ‘The Negative Associations Between Materialism and Pro-Environmental Attitudes and Behaviors: Individual and Regional Evidence From China’, Environment and Behavior, 52(6), pp. 611–638. Available at: https://doi.org/10.1177/0013916518811902.

Hamid, P.N. and Cheng, S.-T. (1995) ‘Predicting Antipollution Behavior: The Role of Molar Behavioral Intentions, Past Behavior, and Locus of Control’, Environment and Behavior, 27(5), pp. 679–698. Available at: https://doi.org/10.1177/0013916595275004.

Hamilton, E.M. (2021) ‘Green Building, Green Behavior? An Analysis of Building Characteristics that Support Environmentally Responsible Behaviors’, Environment and Behavior, 53(4), pp. 409–450. Available at: https://doi.org/10.1177/0013916520942601.

Harris, P.G. (2006) ‘Environmental Perspectives and Behavior in China: Synopsis and Bibliography’, Environment and Behavior, 38(1), pp. 5–21. Available at: https://doi.org/10.1177/0013916505280087.

Hartmann, P. and Apaolaza-Ibáñez, V. (2008) ‘Virtual nature experiences as emotional benefits in green product consumption: The moderating role of environmental attitudes’, Environment and behavior, 40(6), pp. 818–842. Available at: https://doi.org/10.1177/0013916507309870.

Heath, Y. and Gifford, R. (2006) ‘Free-Market Ideology and Environmental Degradation: The Case of Belief in Global Climate Change’, Environment and Behavior, 38(1), pp. 48–71. Available at: https://doi.org/10.1177/0013916505277998.

Heberlein, T.A. and Black, J.S. (1981) ‘Cognitive Consistency and Environmental Action’, Environment and Behavior, 13(6), pp. 717–734. Available at: https://doi.org/10.1177/0013916581136005.

Hedge, A. (1982) ‘The Open-Plan Office: A Systematic Investigation of Employee Reactions to Their Work Environment’, Environment and Behavior, 14(5), pp. 519–542. Available at: https://doi.org/10.1177/0013916582145002.

Hernández, B. et al. (2000) ‘The Study of Environmental Beliefs by Facet Analysis: Research in the Canary Islands, Spain’, Environment and Behavior, 32(5), pp. 612–636. Available at: https://doi.org/10.1177/00139160021972702.

Hood, M.G. (1993) ‘Comfort and Caring: Two Essential Environmental Factors’, Environment and Behavior, 25(6), pp. 710–724. Available at: https://doi.org/10.1177/0013916593256003.

Horsley, A.D. (1977) ‘The Effects of a Social Learning Experiment on Attitudes and Behavior toward Environmental Conservation’, Environment and Behavior, 9(3), pp. 349–384. Available at: https://doi.org/10.1177/001391657700900304.

Howell, A.P., Shaw, B.R. and Alvarez, G. (2015) ‘Bait Shop Owners as Opinion Leaders: A Test of the Theory of Planned Behavior to Predict Pro-Environmental Outreach Behaviors and Intentions’, Environment and Behavior, 47(10), pp. 1107–1126. Available at: https://doi.org/10.1177/0013916514539684.

Ignatow, G. (2006) ‘Cultural Models of Nature and Society: Reconsidering Environmental Attitudes and Concern’, Environment and Behavior, 38(4), pp. 441–461. Available at: https://doi.org/10.1177/0013916505280791.

Johns, K.D., Khovanova, K.M. and Welch, E.W. (2009) ‘Fleet Conversion in Local Government: Determinants of Driver Fuel Choice for Bi-Fuel Vehicles’, Environment and Behavior, 41(3), pp. 402–426. Available at: https://doi.org/10.1177/0013916507312423.

Johnson, C.Y., Bowker, J.M. and Cordell, H.K. (2004) ‘Ethnic Variation in Environmental Belief and Behavior: An Examination of the New Ecological Paradigm in a Social Psychological Context’, Environment and Behavior, 36(2), pp. 157–186. Available at: https://doi.org/10.1177/0013916503251478.

Johnson, E.W. and Schwadel, P. (2019) ‘It Is Not a Cohort Thing: Interrogating the Relationship Between Age, Cohort, and Support for the Environment’, Environment and Behavior, 51(7), pp. 879–901. Available at: https://doi.org/10.1177/0013916518780483.

Johnson, R.J. and Scicchitano, M.J. (2012) ‘Don’t Call Me NIMBY: Public Attitudes Toward Solid Waste Facilities’, Environment and Behavior, 44(3), pp. 410–426. Available at: https://doi.org/10.1177/0013916511435354.

Joireman, J.A., Van Lange, P.A. and Van Vugt, M. (2004) ‘Who cares about the environmental impact of cars? Those with an eye toward the future’, Environment and behavior, 36(2), pp. 187–206. Available at: https://doi.org/10.1177/0013916503251476.

Jones, R.E., Davis, K.L. and Bradford, J. (2013) ‘The Value of Trees: Factors Influencing Homeowner Support for Protecting Local Urban Trees’, Environment and Behavior, 45(5), pp. 650–676. Available at: https://doi.org/10.1177/0013916512439409.

Jorgenson, A.K. and Givens, J.E. (2014) ‘Economic Globalization and Environmental Concern: A Multilevel Analysis of Individuals Within 37 Nations’, Environment and Behavior, 46(7), pp. 848–871. Available at: https://doi.org/10.1177/0013916513479796.

Kahlor, L.A. et al. (2020) ‘Avoiding Trouble: Exploring Environmental Risk Information Avoidance Intentions.’, Environment and Behavior, 52(2), pp. 187–218. Available at: https://doi.org/10.1177/0013916518799149.

Kahn, P.H. and Lourenço, O. (2002) ‘Water, Air, Fire, and Earth: A Developmental Study in Portugal of Environmental Moral Reasoning’, Environment and Behavior, 34(4), pp. 405–430. Available at: https://doi.org/10.1177/00116502034004001.

Kaiser, F.G. et al. (2013) ‘Environmental Protection and Nature as Distinct Attitudinal Objects: An Application of the Campbell Paradigm’, Environment and Behavior, 45(3), pp. 369–398. Available at: https://doi.org/10.1177/0013916511422444.

Kaklamanou, D. et al. (2015) ‘Using public transport can make up for flying abroad on holiday: compensatory green beliefs and environmentally significant behavior’, Environment and Behavior, 47(2), pp. 184–204. Available at: https://doi.org/10.1177/0013916513488784.

Kentmen Cin, C. (2013) ‘Blaming the Government for Environmental Problems: A Multilevel and Cross-National Analysis of the Relationship Between Trust in Government and Local and Global Environmental Concerns’, Environment and Behavior, 45(8), pp. 971–992. Available at: https://doi.org/10.1177/0013916512453840.

Keuschnigg, M. and Kratz, F. (2018) ‘Thou Shalt Recycle: How Social Norms of Environmental Protection Narrow the Scope of the Low-Cost Hypothesis’, Environment and Behavior, 50(10), pp. 1059–1091. Available at: https://doi.org/10.1177/0013916517726569.

Kilbourne, W.E. et al. (2001) ‘A Multinational Examination of the Role of the Dominant Social Paradigm in Environmental Attitudes of University Students’, Environment and Behavior, 33(2), pp. 209–228. Available at: https://doi.org/10.1177/00139160121972954.

Kurz, T., Linden, M. and Sheehy, N. (2007) ‘Attitudinal and Community Influences on Participation in New Curbside Recycling Initiatives in Northern Ireland’, Environment and Behavior, 39(3), pp. 367–391. Available at: https://doi.org/10.1177/0013916506294152.

Lacasse, K. (2015) ‘The Importance of Being Green: The Influence of Green Behaviors on Americans’ Political Attitudes Toward Climate Change’, Environment and Behavior, 47(7), pp. 754–781. Available at: https://doi.org/10.1177/0013916513520491.

Lacasse, K. (2019) ‘Can’t Hurt, Might Help: Examining the Spillover Effects From Purposefully Adopting a New Pro-Environmental Behavior’, Environment and Behavior, 51(3), pp. 259–287. Available at: https://doi.org/10.1177/0013916517748164.

Laidley, T.M. (2013) ‘The Influence of Social Class and Cultural Variables on Environmental Behaviors: Municipal-Level Evidence From Massachusetts’, Environment and Behavior, 45(2), pp. 170–197. Available at: https://doi.org/10.1177/0013916511416647.

Lam, S.-P. and Chen, J.-K. (2006) ‘What makes customers bring their bags or buy bags from the shop? A survey of customers at a Taiwan hypermarket’, Environment and Behavior, 38(3), pp. 318–332. Available at: https://doi.org/10.1177/0013916505278327.

Lam, S.-P. and Cheng, S.-I. (2002) ‘Cross-Informant Agreement in Reports of Environmental Behavior and the Effect of Cross-Questioning on Report Accuracy’, Environment and Behavior, 34(4), pp. 508–520. Available at: https://doi.org/10.1177/00116502034004006.

Langenbach, B.P. et al. (2020) ‘Cognitive Resources Moderate the Relationship Between Pro-Environmental Attitudes and Green Behavior’, Environment and Behavior, 52(9), pp. 979–995. Available at: https://doi.org/10.1177/0013916519843127.

Larson, K.L., Ibes, D.C. and White, D.D. (2011) ‘Gendered Perspectives About Water Risks and Policy Strategies: A Tripartite Conceptual Approach’, Environment and Behavior, 43(3), pp. 415–438. Available at: https://doi.org/10.1177/0013916510365253.

Larson, L.R., Green, G.T. and Castleberry, S.B. (2011) ‘Construction and Validation of an Instrument to Measure Environmental Orientations in a Diverse Group of Children’, Environment and Behavior, 43(1), pp. 72–89. Available at: https://doi.org/10.1177/0013916509345212.

Lee, P.-S. et al. (2020) ‘Using Episodic Future Thinking to Pre-Experience Climate Change Increases Pro-Environmental Behavior’, Environment and Behavior, 52(1), pp. 60–81. Available at: https://doi.org/10.1177/0013916518790590.

Levi, D. and Kocher, S. (1995) ‘The Spotted Owl Controversy and the Sustainability of Rural Communities in the Pacific Northwest’, Environment and Behavior, 27(5), pp. 631–649. Available at: https://doi.org/10.1177/0013916595275002.

Levi, D., Kocher, S. and Aboud, R. (2001) ‘Technological Disasters in Natural and Built Environments’, Environment and Behavior, 33(1), pp. 78–92. Available at: https://doi.org/10.1177/00139160121972873.

Li, L.M.W. et al. (2020) ‘The Relationship Between Dialectical Beliefs and Proenvironmental Behaviors’, Environment and Behavior, 52(3), pp. 223–247. Available at: https://doi.org/10.1177/0013916518799821.

Liu, J.H., Bonzon-Liu, B. and Pierce-Guarino, M. (1997) ‘Common Fate between Humans and Animals?: The Dynamical Systems Theory of Groups and Environmental Attitudes in the Florida Keys’, Environment and Behavior, 29(1), pp. 87–122. Available at: https://doi.org/10.1177/001391659702900104.

Loo, C. and Ong, P. (1984) ‘Crowding Perceptions, Attitudes, and Consequences among the Chinese’, Environment and Behavior, 16(1), pp. 55–87. Available at: https://doi.org/10.1177/0013916584161003.

Lubell, M. (2002) ‘Environmental Activism as Collective Action’, Environment and Behavior, 34(4), pp. 431–454. Available at: https://doi.org/10.1177/00116502034004002.

Lutz, A.R., Simpson-Housley, P. and Deman, A.F. (1999) ‘Wilderness: Rural and Urban Attitudes and Perceptions’, Environment and Behavior, 31(2), pp. 259–266. Available at: https://doi.org/10.1177/00139169921972092.

Lyons, E. and Breakwell, G.M. (1994) ‘Factors Predicting Environmental Concern and Indifference in 13- to 16-Year-Olds’, Environment and Behavior, 26(2), pp. 223–238. Available at: https://doi.org/10.1177/001391659402600205.

Macias, T. and Williams, K. (2016) ‘Know Your Neighbors, Save the Planet: Social Capital and the Widening Wedge of Pro-Environmental Outcomes’, Environment and Behavior, 48(3), pp. 391–420. Available at: https://doi.org/10.1177/0013916514540458.

Maggiotto, M.A. and Bowman, A. (1982) ‘Policy Orientations and Environmental Regulation: A Case Study of Florida’s Legislators’, Environment and Behavior, 14(2), pp. 155–170. Available at: https://doi.org/10.1177/0013916584142002.

Martini, N.F. et al. (2015) ‘Why “feed the lawn”? Exploring the influences on residential turf grass fertilization in the Minneapolis- Saint Paul metropolitan area’, Environment and Behavior, 47(2), pp. 158–183. Available at: https://doi.org/10.1177/0013916513492418.

Mazmanian, D. and Sabatier, P. (1981) ‘Liberalism, Environmentalism, and Partisanship in Public Policy-Making: The California Coastal Commissions’, Environment and Behavior, 13(3), pp. 361–384. Available at: https://doi.org/10.1177/0013916581133007.

McFarlane, B.L. and Hunt, L.M. (2006) ‘Environmental Activism in the Forest Sector: Social Psychological, Social-Cultural, and Contextual Effects’, Environment and Behavior, 38(2), pp. 266–285. Available at: https://doi.org/10.1177/0013916505277999.

Meinhold, J.L. and Malkus, A.J. (2005) ‘Adolescent Environmental Behaviors: Can Knowledge, Attitudes, and Self-Efficacy Make a Difference?’, Environment and Behavior, 37(4), pp. 511–532. Available at: https://doi.org/10.1177/0013916504269665.

Milfont, T.L., Duckitt, J. and Cameron, L.D. (2006) ‘A Cross-Cultural Study of Environmental Motive Concerns and Their Implications for Proenvironmental Behavior’, Environment and Behavior, 38(6), pp. 745–767. Available at: https://doi.org/10.1177/0013916505285933.

Mobley, C. (2016) ‘What Matters When Explaining Environmentalism at the Watershed Level: Who You Are, Where You Live, What You See, or What You Perceive?’, Environment and Behavior, 48(9), pp. 1148–1174. Available at: https://doi.org/10.1177/0013916515586058.

Mobley, C., Vagias, W.M. and DeWard, S.L. (2010) ‘Exploring Additional Determinants of Environmentally Responsible Behavior: The Influence of Environmental Literature and Environmental Attitudes’, Environment and Behavior, 42(4), pp. 420–447. Available at: https://doi.org/10.1177/0013916508325002.

Nisbet, E.K., Zelenski, J.M. and Murphy, S.A. (2009) ‘The Nature Relatedness Scale: Linking Individuals’ Connection With Nature to Environmental Concern and Behavior’, Environment and Behavior, 41(5), pp. 715–740. Available at: https://doi.org/10.1177/0013916508318748.

Nixon, H. et al. (2009) ‘Understanding Preferences for Recycling Electronic Waste in California: The Influence of Environmental Attitudes and Beliefs on Willingness to Pay’, Environment and Behavior, 41(1), pp. 101–124. Available at: https://doi.org/10.1177/0013916507310053.

Nooney, J.G. et al. (2003) ‘Environmental Worldview and Behavior: Consequences of Dimensionality in a Survey of North Carolinians’, Environment and Behavior, 35(6), pp. 763–783. Available at: https://doi.org/10.1177/0013916503256246.

Ojala, M. (2008) ‘Recycling and Ambivalence: Quantitative and Qualitative Analyses of Household Recycling Among Young Adults’, Environment and Behavior, 40(6), pp. 777–797. Available at: https://doi.org/10.1177/0013916507308787.

Olli, E., Grendstad, G. and Wollebaek, D. (2001) ‘Correlates of Environmental Behaviors: Bringing Back Social Context’, Environment & Behavior, 33(2), pp. 181–208. Available at: https://doi.org/10.1177/00139160121972945.

Olofsson, A. and Öhman, S. (2006) ‘General Beliefs and Environmental Concern: Transatlantic Comparisons’, Environment and Behavior, 38(6), pp. 768–790. Available at: https://doi.org/10.1177/0013916506287388.

Oreg, S. and Katz-Gerro, T. (2006) ‘Predicting Proenvironmental Behavior Cross-Nationally: Values, the Theory of Planned Behavior, and Value-Belief-Norm Theory’, Environment and Behavior, 38(4), pp. 462–483. Available at: https://doi.org/10.1177/0013916505286012.

Oskamp, S. et al. (1991) ‘Factors Influencing Household Recycling Behavior’, Environment and Behavior, 23(4), pp. 494–519. Available at: https://doi.org/10.1177/0013916591234005.

Pahl, S. and Bauer, J. (2013) ‘Overcoming the Distance: Perspective Taking With Future Humans Improves Environmental Engagement’, Environment and Behavior, 45(2), pp. 155–169. Available at: https://doi.org/10.1177/0013916511417618.

Panno, A. et al. (2018) ‘Mindfulness, Pro-environmental Behavior, and Belief in Climate Change: The Mediating Role of Social Dominance’, Environment and Behavior, 50(8), pp. 864–888. Available at: https://doi.org/10.1177/0013916517718887.

Parant, A. et al. (2017) ‘Raising Students Awareness to Climate Change: An Illustration With Binding Communication’, Environment and Behavior, 49(3), pp. 339–353. Available at: https://doi.org/10.1177/0013916516629191.

Parker, J.D. and McDonough, M.H. (1999) ‘Environmentalism of African Americans: An Analysis of the Subculture and Barriers Theories’, Environment and Behavior, 31(2), pp. 155–177. Available at: https://doi.org/10.1177/00139169921972047.

Pelletier, L.G., Legault, L.R. and Tuson, K.M. (1996) ‘The Environmental Satisfaction Scale: A Measure of Satisfaction with Local Environmental Conditions and Government Environmental Policies’, Environment and Behavior, 28(1), pp. 5–26. Available at: https://doi.org/10.1177/0013916596281001.

Pisano, I. and Lubell, M. (2017) ‘Environmental Behavior in Cross-National Perspective: A Multilevel Analysis of 30 Countries’, Environment and Behavior, 49(1), pp. 31–58. Available at: https://doi.org/10.1177/0013916515600494.

Pooley, J.A. and O’Connor, M. (2000) ‘Environmental Education and Attitudes: Emotions and Beliefs are What is Needed’, Environment and Behavior, 32(5), pp. 711–723. Available at: https://doi.org/10.1177/0013916500325007.

Poortinga, W., Steg, L. and Vlek, C. (2002) ‘Environmental Risk Concern and Preferences for Energy-Saving Measures’, Environment and Behavior, 34(4), pp. 455–478. Available at: https://doi.org/10.1177/00116502034004003.

Poortinga, W., Steg, L. and Vlek, C. (2004) ‘Values, Environmental Concern, and Environmental Behavior: A Study into Household Energy Use’, Environment and Behavior, 36(1), pp. 70–93. Available at: https://doi.org/10.1177/0013916503251466.

Prati, G. and Zani, B. (2013) ‘The Effect of the Fukushima Nuclear Accident on Risk Perception, Antinuclear Behavioral Intentions, Attitude, Trust, Environmental Beliefs, and Values’, Environment and Behavior, 45(6), pp. 782–798. Available at: https://doi.org/10.1177/0013916512444286.

Puech, C. et al. (2020) ‘Openness Is Related to Proenvironmental Behavior Both Within and Across Families’, Environment and Behavior, 52(9), pp. 996–1011. Available at: https://doi.org/10.1177/0013916519853294.

Qian, C., Yu, K. and Gao, J. (2021) ‘Understanding Environmental Attitude and Willingness to Pay With an Objective Measure of Attitude Strength’, Environment and Behavior, 53(2), pp. 119–150. Available at: https://doi.org/10.1177/0013916519855140.

Rauwald, K.S. and Moore, C.F. (2002) ‘Environmental Attitudes as Predictors of Policy Support across Three Countries’, Environment and Behavior, 34(6), pp. 709–739. Available at: https://doi.org/10.1177/001391602237243.

Ribe, R.G. (2002) ‘Is Scenic Beauty a Proxy for Acceptable Management?: The Influence of Environmental Attitudes on Landscape Perceptions’, Environment and Behavior, 34(6), pp. 757–780. Available at: https://doi.org/10.1177/001391602237245.

Roccato, M., Mannarini, T. and Pacilli, M.G. (2018) ‘Ingroup Identification, Outgroup Infrahumanization, and Intention to Mobilize in Land Use Conflicts’, Environment and Behavior, 50(5), pp. 512–534. Available at: https://doi.org/10.1177/0013916517708396.

Roczen, N. et al. (2014) ‘A Competence Model for Environmental Education’, Environment and Behavior, 46(8), pp. 972–992. Available at: https://doi.org/10.1177/0013916513492416.

Rogers, M. et al. (2015) ‘Water Management and Healthy Ageing in Rural Australia: Economic, Social, and Cultural Considerations’, Environment and Behavior, 47(5), pp. 551–569. Available at: https://doi.org/10.1177/0013916513502355.

Rosentrater, L.D. et al. (2013) ‘Efficacy Trade-Offs in Individuals’ Support for Climate Change Policies’, Environment and Behavior, 45(8), pp. 935–970. Available at: https://doi.org/10.1177/0013916512450510.

Ruiz, C., Marrero, R. and Hernández, B. (2018) ‘Influence of Emotions on the Acceptance of an Oil Drilling Project’, Environment and Behavior, 50(3), pp. 324–349. Available at: https://doi.org/10.1177/0013916517701795.

Ryan, R.L. (2005) ‘Exploring the Effects of Environmental Experience on Attachment to Urban Natural Areas’, Environment and Behavior, 37(1), pp. 3–42. Available at: https://doi.org/10.1177/0013916504264147.

Sachdeva, S. (2017) ‘The Influence of Sacred Beliefs in Environmental Risk Perception and Attitudes’, Environment and Behavior, 49(5), pp. 583–600. Available at: https://doi.org/10.1177/0013916516649413.

Sarigöllü, E. (2009) ‘A Cross-Country Exploration of Environmental Attitudes’, Environment and Behavior, 41(3), pp. 365–386. Available at: https://doi.org/10.1177/0013916507313920.

Schahn, J. and Holzer, E. (1990) ‘Studies of Individual Environmental Concern: The Role of Knowledge, Gender, and Background Variables’, Environment and Behavior, 22(6), pp. 767–786. Available at: https://doi.org/10.1177/0013916590226003.

Schultz, P.W. and Stone, W.F. (1994) ‘Authoritarianism and Attitudes Toward the Environment’, Environment and Behavior, 26(1), pp. 25–37. Available at: https://doi.org/10.1177/0013916594261002.

Schultz, P.W., Zelezny, L. and Dalrymple, N.J. (2000) ‘A Multinational Perspective on the Relation between Judeo-Christian Religious Beliefs and Attitudes of Environmental Concern’, Environment and Behavior, 32(4), pp. 576–591. Available at: https://doi.org/10.1177/00139160021972676.

Scott, D. (1999) ‘Equal Opportunity, Unequal Results: Determinants of Household Recycling Intensity’, Environment and Behavior, 31(2), pp. 267–290. Available at: https://doi.org/10.1177/00139169921972100.

Scott, D. and Willits, F.K. (1994) ‘Environmental Attitudes and Behavior: A Pennsylvania Survey’, Environment and Behavior, 26(2), pp. 239–260. Available at: https://doi.org/10.1177/001391659402600206.

Sebba, R. (1991) ‘The Landscapes of Childhood: The Reflection of Childhood’s Environment in Adult Memories and in Children’s Attitudes’, Environment and Behavior, 23(4), pp. 395–422. Available at: https://doi.org/10.1177/0013916591234001.

Seguin, C., Pelletier, L.G. and Hunsley, J. (1998) ‘Toward a Model of Environmental Activism’, Environment and Behavior, 30(5), pp. 628–652. Available at: https://doi.org/10.1177/001391659803000503.

Sewell, D.W.R. (1971) ‘Environmental Perceptions and Attitudes of Engineers and Public Health Officials’, Environment and Behavior, 3(1), pp. 23–59. Available at: https://doi.org/10.1177/001391657100300102.

deHaven-Smith, L. (1988) ‘Environmental Belief Systems: Public Opinion on Land Use Regulation in Florida’, Environment and Behavior, 20(3), pp. 276–299. Available at: https://doi.org/10.1177/0013916588203002.

Staats, H., Harland, P. and Wilke, H.A.M. (2004) ‘Effecting Durable Change: A Team Approach to Improve Environmental Behavior in the Household’, Environment and Behavior, 36(3), pp. 341–367. Available at: https://doi.org/10.1177/0013916503260163.

Stahl, A. (1993) ‘Educating for Change in Attitudes toward Nature and Environment among Oriental Jews in Israel’, Environment and Behavior, 25(1), pp. 3–21. Available at: https://doi.org/10.1177/0013916593251001.

Steg, L. et al. (2014) ‘The Significance of Hedonic Values for Environmentally Relevant Attitudes, Preferences, and Actions’, Environment and Behavior, 46(2), pp. 163–192. Available at: https://doi.org/10.1177/0013916512454730.

Stern, P.C., Dietz, T. and Guagnano, G.A. (1995) ‘The New Ecological Paradigm in Social-Psychological Context’, Environment and Behavior, 27(6), pp. 723–743. Available at: https://doi.org/10.1177/0013916595276001.

Syme, G.J., Nancarrow, B.E. and Jorgensen, B.S. (2002) ‘The Limits of Environmental Responsibility: A Stormwater Case Study’, Environment and Behavior, 34(6), pp. 836–847. Available at: https://doi.org/10.1177/001391602237251.

Takahashi, B. and Selfa, T. (2015) ‘Predictors of Pro-Environmental Behavior in Rural American Communities’, Environment and Behavior, 47(8), pp. 856–876. Available at: https://doi.org/10.1177/0013916514521208.

Takahashi, L.M. and Gaber, S.L. (1998) ‘Controversial Facility Siting in the Urban Environment: Resident and Planner Perceptions in the United States’, Environment and Behavior, 30(2), pp. 184–215. Available at: https://doi.org/10.1177/0013916598302004.

Tang, Z., Chen, X. and Luo, J. (2011) ‘Determining Socio-Psychological Drivers for Rural Household Recycling Behavior in Developing Countries: A Case Study From Wugan, Hunan, China’, Environment and Behavior, 43(6), pp. 848–877. Available at: https://doi.org/10.1177/0013916510375681.

Tarrant, M.A. and Cordell, H.K. (1997) ‘The Effect of Respondent Characteristics on General Environmental Attitude-Behavior Correspondence’, Environment and Behavior, 29(5), pp. 618–637. Available at: https://doi.org/10.1177/0013916597295002.

Teisl, M.F. and O’Brien, K. (2003) ‘Who cares and who acts? Outdoor recreationists exhibit different levels of environmental concern and behavior’, Environment and behavior, 35(4), pp. 506–522. Available at: https://doi.org/10.1177/0013916503251461.

Thomas, G.O. and Walker, I. (2016) ‘The Development and Validation of an Implicit Measure Based on Biospheric Values’, Environment and Behavior, 48(5), pp. 659–685. Available at: https://doi.org/10.1177/0013916514553836.

Tobias, R., Brügger, A. and Mosler, H.-J. (2009) ‘Developing Strategies for Waste Reduction by Means of Tailored Interventions in Santiago de Cuba’, Environment and Behavior, 41(6), pp. 836–865. Available at: https://doi.org/10.1177/0013916509338004.

Toner, K., Gan, M. and Leary, M.R. (2014) ‘The Impact of Individual and Group Feedback on Environmental Intentions and Self-Beliefs’, Environment and Behavior, 46(1), pp. 24–45. Available at: https://doi.org/10.1177/0013916512451902.

Truelove, H.B. and Joireman, J. (2009) ‘Understanding the Relationship Between Christian Orthodoxy and Environmentalism: The Mediating Role of Perceived Environmental Consequences’, Environment and Behavior, 41(6), pp. 806–820. Available at: https://doi.org/10.1177/0013916508328905.

Tzamir, Y. and Churchman, A. (1984) ‘Knowledge, Ethics, and Environment: Behavior Studies in Architectural Education’, Environment and Behavior, 16(1), pp. 111–126. Available at: https://doi.org/10.1177/0013916584161005.

Urban, J., Braun Kohlová, M. and Bahník, Š. (2021) ‘No Evidence of Within-Domain Moral Licensing in the Environmental Domain’, Environment and Behavior, 53(10), pp. 1070–1094. Available at: https://doi.org/10.1177/0013916520942604.

Uzzell, D., Pol, E. and Badenas, D. (2002) ‘Place Identification, Social Cohesion, and Enviornmental Sustainability’, Environment and Behavior, 34(1), pp. 26–53. Available at: https://doi.org/10.1177/0013916502034001003.

Valera, S. and Guàrdia, J. (2002) ‘Urban Social Identity and Sustainability: Barcelona’s Olympic Village’, Environment and Behavior, 34(1), pp. 54–66. Available at: https://doi.org/10.1177/0013916502034001004.

Van Birgelen, M., Semeijn, J. and Keicher, M. (2009) ‘Packaging and Proenvironmental Consumption Behavior: Investigating Purchase and Disposal Decisions for Beverages’, Environment and Behavior, 41(1), pp. 125–146. Available at: https://doi.org/10.1177/0013916507311140.

Van Der Pligt, J., Eiser, J.R. and Spears, R. (1986) ‘Attitudes toward Nuclear Energy: Familiarity and Salience’, Environment and Behavior, 18(1), pp. 75–93. Available at: https://doi.org/10.1177/0013916586181004.

Van Riper, C.J. et al. (2020) ‘Values, Motivations, and Intentions to Engage in Proenvironmental Behavior’, Environment and Behavior, 52(4), pp. 437–462. Available at: https://doi.org/10.1177/0013916518807963.

Verges, M. and Duffy, S. (2010) ‘Connected to Birds but Not Bees: Valence Moderates Implicit Associations with Nature’, Environment and Behavior, 42(5), pp. 625–642. Available at: https://doi.org/10.1177/0013916508330210.

Villacorta, M., Koestner, R. and Lekes, N. (2003) ‘Further validation of the motivation toward the environment scale’, Environment and Behavior, 35(4), pp. 486–505. Available at: https://doi.org/10.1177/0013916503035004003.

Vogel, S. (1996) ‘Farmers’ Environmental Attitudes and Behavior: A Case Study for Austria’, Environment and Behavior, 28(5), pp. 591–613. Available at: https://doi.org/10.1177/001391659602800502.

Vorkinn, M. and Riese, H. (2001) ‘Environmental Concern in a Local Context: The Significance of Place Attachment’, Environment and Behavior, 33(2), pp. 249–263. Available at: https://doi.org/10.1177/00139160121972972.

Wang, Y. (2017) ‘Promoting Sustainable Consumption Behaviors: The Impacts of Environmental Attitudes and Governance in a Cross-National Context’, Environment and Behavior, 49(10), pp. 1128–1155. Available at: https://doi.org/10.1177/0013916516680264.

Webber, D.J. (1982) ‘Is Nuclear Power Just Another Environmental Issue?: An Analysis of California Voters’, Environment and Behavior, 14(1), pp. 72–83. Available at: https://doi.org/10.1177/0013916582141004.

Welte, T.H.L. and Anastasio, P.A. (2010) ‘To Conserve or Not to Conserve: Is Status the Question?’, Environment and Behavior, 42(6), pp. 845–863. Available at: https://doi.org/10.1177/0013916509348461.

Wener, R.E. and Kaminoff, R.D. (1983) ‘Improving Environmental Information: Effects of Signs on Perceived Crowding and Behavior’, Environment and Behavior, 15(1), pp. 3–20. Available at: https://doi.org/10.1177/0013916583151001.

Whitburn, J., Linklater, W.L. and Milfont, T.L. (2019) ‘Exposure to Urban Nature and Tree Planting Are Related to Pro-Environmental Behavior via Connection to Nature, the Use of Nature for Psychological Restoration, and Environmental Attitudes’, Environment and Behavior, 51(7), pp. 787–810. Available at: https://doi.org/10.1177/0013916517751009.

Wohlwill, J.F. (1979) ‘The Social and Political Matrix of Environmental Attitudes: An Analysis of the Vote on the California Coastal Zone Regulation Act’, Environment and Behavior, 11(1), pp. 71–85. Available at: https://doi.org/10.1177/0013916579111003.

Wray-Lake, L., Flanagan, C.A. and Osgood, D.W. (2010) ‘Examining Trends in Adolescent Environmental Attitudes, Beliefs, and Behaviors Across Three Decades’, Environment and Behavior, 42(1), pp. 61–85. Available at: https://doi.org/10.1177/0013916509335163.

Wynveen, C.J., Kyle, G.T. and Sutton, S.G. (2014) ‘Environmental Worldview, Place Attachment, and Awareness of Environmental Impacts in a Marine Environment’, Environment and Behavior, 46(8), pp. 993–1017. Available at: https://doi.org/10.1177/0013916513484325.

Wysor, M.S. (1983) ‘Comparing College Students’ Environmental Perceptions and Attitudes: A Methodological Investigation’, Environment and Behavior, 15(5), pp. 615–645. Available at: https://doi.org/10.1177/0013916583155004.

Xiao, C. (2013) ‘Public Attitudes Toward Science and Technology and Concern for the Environment: Testing a Model of Indirect Feedback Effects’, Environment and Behavior, 45(1), pp. 113–137. Available at: https://doi.org/10.1177/0013916511414875.

Xiao, C. and Hong, D. (2018) ‘Gender Differences in Environmental Behaviors Among the Chinese Public: Model of Mediation and Moderation’, Environment and Behavior, 50(9), pp. 975–996. Available at: https://doi.org/10.1177/0013916517723126.

Xiao, C. and McCright, A.M. (2014) ‘A Test of the Biographical Availability Argument for Gender Differences in Environmental Behaviors’, Environment and Behavior, 46(2), pp. 241–263. Available at: https://doi.org/10.1177/0013916512453991.

Xiao, C. and McCright, A.M. (2015) ‘Gender Differences in Environmental Concern: Revisiting the Institutional Trust Hypothesis in the USA’, Environment and Behavior, 47(1), pp. 17–37. Available at: https://doi.org/10.1177/0013916513491571.

Yabiku, S.T., Casagrande, D.G. and Farley-Metzger, E. (2008) ‘Preferences for Landscape Choice in a Southwestern Desert City’, Environment and Behavior, 40(3), pp. 382–400. Available at: https://doi.org/10.1177/0013916507300359.

Youngentob, K. and Hostetler, M. (2005) ‘Is a New Urban Development Model Building Greener Communities?’, Environment and Behavior, 37(6), pp. 731–759. Available at: https://doi.org/10.1177/0013916505275311.

Zelenski, J.M. and Nisbet, E.K. (2014) ‘Happiness and Feeling Connected: The Distinct Role of Nature Relatedness’, Environment and Behavior, 46(1), pp. 3–23. Available at: https://doi.org/10.1177/0013916512451901.

Žukauskienė, R. et al. (2021) ‘“My Words Matter”: The Role of Adolescents in Changing Pro-environmental Habits in the Family’, Environment and Behavior, 53(10), pp. 1140–1162. Available at: https://doi.org/10.1177/0013916520953150.
